# Microglia exit the CNS in spinal root avulsion

**DOI:** 10.1371/journal.pbio.3000159

**Published:** 2019-02-22

**Authors:** Lauren A. Green, Julia C. Nebiolo, Cody J. Smith

**Affiliations:** 1 Department of Biological Sciences, University of Notre Dame, Notre Dame, Indiana, United States of America; 2 Center for Stem Cells and Regenerative Medicine, University of Notre Dame, Notre Dame, Indiana, United States of America; Duke University Medical Center, UNITED STATES

## Abstract

Microglia are central nervous system (CNS)-resident cells. Their ability to migrate outside of the CNS, however, is not understood. Using time-lapse imaging in an obstetrical brachial plexus injury (OBPI) model, we show that microglia squeeze through the spinal boundary and emigrate to peripheral spinal roots. Although both macrophages and microglia respond, microglia are the debris-clearing cell. Once outside the CNS, microglia re-enter the spinal cord in an altered state. These peripheral nervous system (PNS)-experienced microglia can travel to distal CNS areas from the injury site, including the brain, with debris. This emigration is balanced by two mechanisms—induced emigration via *N*-methyl-D-aspartate receptor (NMDA) dependence and restriction via contact-dependent cellular repulsion with macrophages. These discoveries open the possibility that microglia can migrate outside of their textbook-defined regions in disease states.

## Abstract 2

Microglia are normally assumed to be confined to the central nervous system (CNS), but this study shows show that after spinal root injury, microglia can exit the CNS to clear debris. Upon re-entry, the emigrated microglia are altered and can travel to distal areas such as the brain.

## Author summary

Cells are precisely organized in specific anatomical domains to ensure normal functioning of the nervous system. One such cell type, microglia, is usually considered to be confined to the central nervous system (CNS). Using time-lapse imaging to capture microglia as they migrate, we show that their characteristic CNS-residency can be altered after spinal root injury. After such injury, the microglia exit the spinal root to the periphery, where they clear debris at the injury site and then carry that debris back into the CNS. In addition, microglia that leave the CNS after spinal root injury become distinct from those that remain within the CNS. This emigration event of microglia after injury is driven by two mechanisms—dependence on glutamatergic signaling that induces their emigration to the injury and interactions with macrophages that prevent their ectopic exit from the spinal cord. Together, these discoveries raise the possibility that microglia could override their CNS-residency in certain disease contexts.

## Introduction

Microglia are the surveying phagocytic cells of the central nervous system (CNS) [1–3]. They enter the CNS during embryonic development [4,5]. Once in the CNS, their roles include a growing list of functions, including synaptic pruning and clearance of debris from both developmental and injured cells [6–9]. During these processes, microglia transition from a surveying to activated state, leading to increased cellular migration and phagocytic activity [10–12]. This microglia activation can have lasting impacts on the nervous system. For example, pollutants in pregnant mice can lead to altered microglia in their progeny; such embryonic changes are implicated to autism-like phenotypes [13]. Meanwhile, activation after spinal cord injury is linked to neuropathic pain [14]. Despite these growing contributions of microglia to the CNS, little is known about their role outside of the CNS domain.

There is growing evidence that cells can override their domain-specific nature [15–18]. CNS-resident cells like oligodendrocytes can reside in the peripheral nervous system (PNS) in peripheral neuropathy [15]. Similarly, both oligodendrocytes and astrocytes can populate the PNS when boundary cells are disrupted [15–18]. These ectopically localized CNS cells migrate to the PNS from the CNS instead of differentiating from resident PNS cells [16,17]. Despite these examples of CNS cell intrusion, microglial emigration to the PNS is not understood. In each example of ectopic CNS cell residence, the ability of the emigrated cells to return to their respective domains is not known. Given the highly migratory nature of microglia, their emerging roles in circuit formation and maintenance in both healthy and disease states and their inclusions in disorders such as autism, spinal cord injury, neuropathic pain, and multiple sclerosis [12,13], it is imperative to investigate not only the full capacity of microglia to migrate to specific domains but the consequence of such movements. Complicating this issue, microglia and macrophages are labeled with similar molecular markers and when located outside of their resident domain, can express the limited number of specific markers that label either microglia or macrophages.

Here, we exploit time-lapse imaging of zebrafish in a laser-induced model of obstetrical brachial plexus injury (OBPI) to dissect the capacity of microglia in the PNS. Spinal root avulsion can occur developmentally during the birth process; OBPI is a complication in an estimated 3 out of every 1,000 births [19]. We first demonstrate that *pu1*^*+*^ cells display stable residency in distinct CNS and PNS domains at 4 days post fertilization (dpf) in zebrafish, a time comparable to when OBPI occurs. We then show that CNS-resident microglia exit their domain to clear PNS debris in these injuries. Despite that both macrophages and microglia respond to these injuries, microglia function as the debris-clearing cell in both the PNS and CNS. This emigration to the PNS alters the microglia as they re-enter the CNS and migrate to distal areas from the injury site, including the brain. We show that the exit of microglia to the PNS is mediated by opposing mechanisms; *N*-methyl-D-aspartate (NMDA) receptor and glutamate induce emigration, whereas contact-dependent repulsion prevents intrusion. The observation that contact-dependent interactions of microglia with macrophages impact the cells’ anatomical position could provide insight into pathologies of diseases that contain both macrophages and microglia in the same domain [20]. Together, these data provide evidence that microglia function expands beyond their textbook-defined CNS-resident domain.

## Results

### Laser-induced injury model mimics OBPI

**Fig 1:**
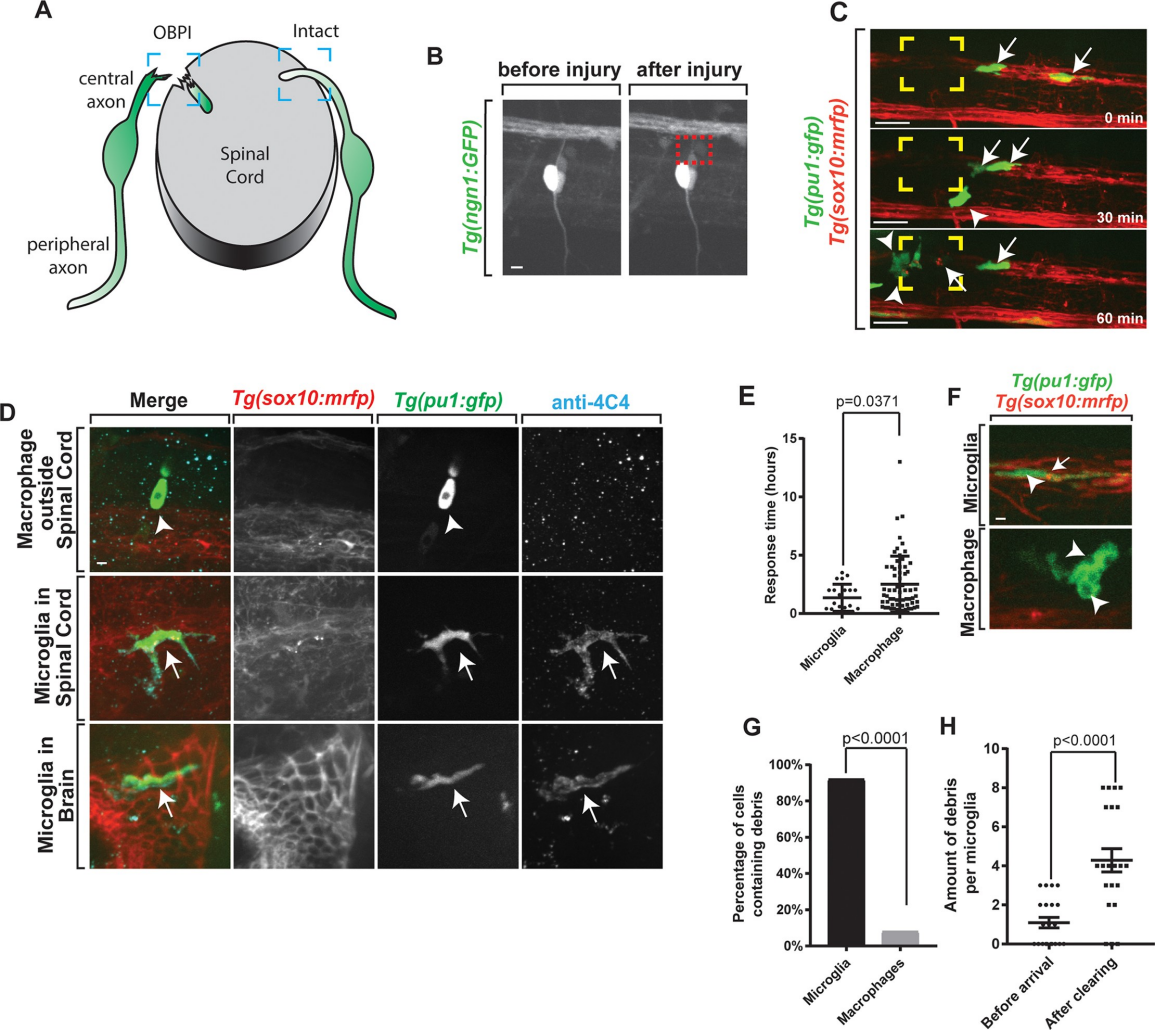
Modeling OBPI. (A) Graphical representation of OBPI model. Boxes indicate injured and intact DREZ. (B) Confocal z-projection of *Tg(ngn1*:*gfp)* zebrafish at 4 dpf showing successful spinal root avulsion. (C) Representative images from 24-hour time-lapse movies starting at 4 dpf in *Tg(pu1*:*gfp);Tg(sox10*:*mrfp)* zebrafish showing the arrival of both microglia and macrophages to the injury site. Arrows indicate microglia. Arrowheads indicate macrophages. Yellow box indicates injury site. (D) Confocal z-projection of *Tg(pu1*:*gfp);Tg(sox10*:*mrfp)* stained with the microglia-specific anti-4C4 antibody showing the presence of microglia in the brain and spinal cord and lack of 4C4 staining in macrophages outside the brain and spinal cord. Arrows indicate microglia. Arrowheads indicate macrophages. (E) Quantification of individual cell response time to site of injury. (F) Images from a 24-hour time-lapse movie starting at 4 dpf in *Tg(pu1*:*gfp);Tg(sox10*:*mrfp)* zebrafish showing debris and vacuoles in phagocytic cells. Arrows indicate *mRFP*^*+*^ debris from *sox10* cells. Arrowheads indicate vacuoles. (G) Quantification of the percentage of microglia and macrophages that contain debris (*p* < 0.0001). (H) Quantification of amount of individual debris puncta within microglia before and after arrival to injury site. Scale bar equals 1 μm (D,F), 10 μm (B,C). Statistics summarized in S1 Table. See S1 Data for raw data. DREZ, dorsal root entry zone; OBPI, obstetrical branchial plexus injury.

To model obstetrical root avulsion, we created injuries in 4 dpf zebrafish. At 4 dpf, zebrafish have an established anatomical organization of neurons in the brain and spinal cord but myelination is ongoing, similar to newborn children (Fig 1A). Cells that compose the spinal sensory root are organized by 2–3 dpf, before our avulsion model at 4 dpf [21,22]. These injuries were created by exposing a 4 μm region of the spinal cord sensory root nerve to pulses of a laser in *Tg(ngn1*:*gfp)* zebrafish, which use regulatory sequences of *ngn1* to express green fluorescent protein (GFP) in dorsal root ganglia (DRG) neurons [21,23] (S1 Movie). To confirm that the laser induced root avulsion, we first created intensity surface plots along DRG projections and measured an absence of intensity in the afferent projection specifically where the laser was exposed (Fig 1B, S1A and S1B Fig). The absence of signal was initially restricted to a small region until the axonal region degraded, leaving a DRG cell soma without a central projection (Fig 1B). This decrease was specific to the lesion site, persisted for hours, and was not created when the peripheral projection was injured (S1C and S1D Fig). In this avulsion model, as with obstetrical avulsions, we created injuries of varying severity (S2A–S2D Fig). We also fixed and stained *Tg(ngn1*:*gfp)* animals at 4 dpf with anti-Sox10 and anti-GFAP post-avulsion to assess the integrity of the GFAP^+^ glial limitans and Sox10^+^ Schwann cells and oligodendrocytes in each injury category immediately following injury (S3A Fig). Glial fibrillary acidic protein (GFAP) fluorescently labels the glial limitans, or the radial glial boundary. DRG cell bodies and supporting *Sox10*^*+*^ cell nuclei were present and intact in uninjured animals and category I and II injuries (S3A Fig). The *GFAP*^*+*^ boundary was disrupted in category III injuries with little damage in category I–II (S3B–S3E Fig). These data recapitulate characteristics of spinal avulsion with varying degrees of severity but demonstrate that category I and II injuries lack massive damage of the spinal interface.

### Microglia in the spinal cord respond to OBPI

In movies of these injuries, we noted that neural debris of both glial and neuronal identity was present in both CNS and PNS regions. To investigate debris clearance, we visualized phagocytic cells in response to injury [24] (Fig 1C, S2 Movie). To do this, we imaged *Tg(pu1*:*gfp); Tg(sox10*:*mrfp)* animals, which use regulatory sequences of *pu1* to express GFP in microglial and macrophages and *sox10* to label glial and neuronal cells of sensory and spinal nerves with mRFP [25,26]. With this imaging, we first distinguished between *pu1*^*+*^ cells like PNS macrophages and CNS microglia based on their stable starting location without injury (Fig 1C, S4A and S4B Fig). By definition, microglia are stable CNS-resident cells that are labeled with *pu1* and anti-4C4 and are sensitive to *csf1r* inhibitors (S4C Fig). To confirm their identity, before injuries, we stained for 4C4, which labels microglia but not macrophages in zebrafish [27], and detected 4C4 overlap only with CNS-located *pu1*^+^ cells and not peripheral cells (Fig 1D). These spinal cord-located 4C4^+^;*pu1*^+^ cells resembled well-defined microglia in the brain [5,27] (Fig 1D). Additionally, we assayed for the microglia-specific transcript *tmem119* [28] with a smFISH probes and detected colocalization with *pu1*^*+*^ *microglia* in the spinal cord (S4F Fig). To confirm microglia in the spinal cord at 4 dpf, we then tested that in noninjured animals, *pu1*^*+*^ cells in the CNS and PNS maintain their domain-specific residency. In confocal images of the spinal cord, *pu1*^*+*^ cells in the spinal cord could first be detected at 3.5 dpf. The number of *pu1*^*+*^ cells in the spinal cord then increased during development. To determine their stable residency, time-lapse imaging from 4–5 dpf was used in noninjured animals. Consistent with their identity as microglia, *pu1*^*+*^ cells in the spinal cord at 4 dpf remained resident in the CNS (100% of microglia remained in the spinal cord, *n* = 5 animals). These movies revealed that the majority of *pu1*^*+*^ cells that colonize the spinal cord at 4 dpf originate from the anterior, potentionally the brain region, which colonizes microglia by 2.5 dpf [26]. Not only were CNS cells marked by *pu1*^*+*^, 4C4, and *tmem119*, they were also located in the spinal cord proper, distal from the spinal meninges space and the spinal vascular network, and were sensitive to *csf1r* inhibitors, consistent with their classification as microglia (S4C, S4E and S4G Fig) [2]. PNS-located *pu1*^*+*^ cells were similarly stable in their PNS anatomical domain in 24-hour movies. These data are consistent with the hypothesis that the domain residency of *pu1*^*+*^ cells like CNS-resident microglia and PNS-resident macrophages are established by 4 dpf in zebrafish, a comparable developmental time to when OPBIs occur.

### Microglia are the primary debris clearing cell at injury sites

To dissect phagocytic cellular response in OBPI, we then created injuries in *Tg(pu1*:*gfp); Tg(sox10*:*mrfp)* animals. After injury, we imaged z-stacks that spanned the root and spinal cord every 2.5 minutes for 24 hours. Following spinal avulsion and consistent with a microglial injury response, we visualized microglia migrated to the injury site within the first hour following injury (Fig 1E, S5A Fig) [29,30]. In these movies, we also visualized macrophages responding immediately to the injury, traveling at an average velocity of 151.34 μm/h compared to microglia, which respond at a velocity of 203.58 μm/h (S5B Fig). To ask whether these responses were correlated with the size of the injury, we categorized injuries based on the lesion size (S5C and S5D Fig). However, all size injuries provoked microglial response (S5C and S5D Fig). To determine the migration path of these cells, we tracked individual cells and found that the migration path of microglia and macrophages was direct (S5E Fig). Microglia traveled 72.72 μm to injury compared to macrophages traveling 58.60 μm on average, with different velocities (S5B and S5F Fig). Given that both CNS and PNS cells responded, we next asked which cells responded first by quantifying the percentage of injuries for which each cell was a first responder. Despite the difference in distance traveled, microglia and macrophages each were first responders 50% of the time (S5G Fig). Although macrophages outnumbered microglia at the injury site nearly 3-fold. These data are consistent with the hypothesis that both microglia and macrophages respond to spinal root avulsion.

Because both cells responded to the injury, we next tested whether, while at the injury site, microglia and macrophages each clear debris. To do this, we injured *Tg(pu1*:*gfp); Tg(sox10*:*mrfp)* animals, created 24-hour movies, identified CNS and PNS GFP^+^ cells based on their pre-injury location, and then scored mRFP debris within GFP^+^ cells, a result consistent with clearance of debris from GFP^+^ cells [26,31,32]. Although both macrophages and microglia responded to injury, mRFP^+^ debris was present in 92% of microglia, whereas mRFP^+^ debris was present in 8% of macrophages (Fig 1F and 1G), consistent with the hypothesis that microglia primarily clear debris. We confirmed that the phagocytosis of mRFP^+^ debris from microglia was specific to the injury site by scoring an average of 1.2 mRFP^+^ debris puncta before microglia arrived to the injury site, which increased to 4.3 mRFP^+^ debris after arrival (Fig 1H). Although mRFP^+^ debris was primarily in microglia, phagocytic vacuoles could be seen in both macrophages and microglia, consistent with the previous hypotheses that both cells are capable of phagocytosis (S6A and S6B Fig). These vacuoles were specific to the injury site, because after arrival to injury, on average 2.18 vacuoles were present compared to the 0 vacuoles before arrival (S6A and S6B Fig). Although the vacuoles lacked mRFP^+^ debris in macrophages, every microglial cell with vacuoles contained mRFP^+^ debris. If microglia are the primary debris-clearing cells in OBPI, we hypothesized that they would clear debris longer at the injury side than macrophages. To test this, we tracked the distinct *pu1*^*+*^ cell populations in time-lapse movies and scored microglia spend on average 9.14 hours at the injury site compared to macrophages, which spend 5.97 hours (S6C and S6D Fig), a result that is consistent with the hypothesis that microglia could function as the primary debris-clearing cells following obstetrical avulsion.

### Microglia exit the CNS during spinal root avulsion

**Fig 2:**
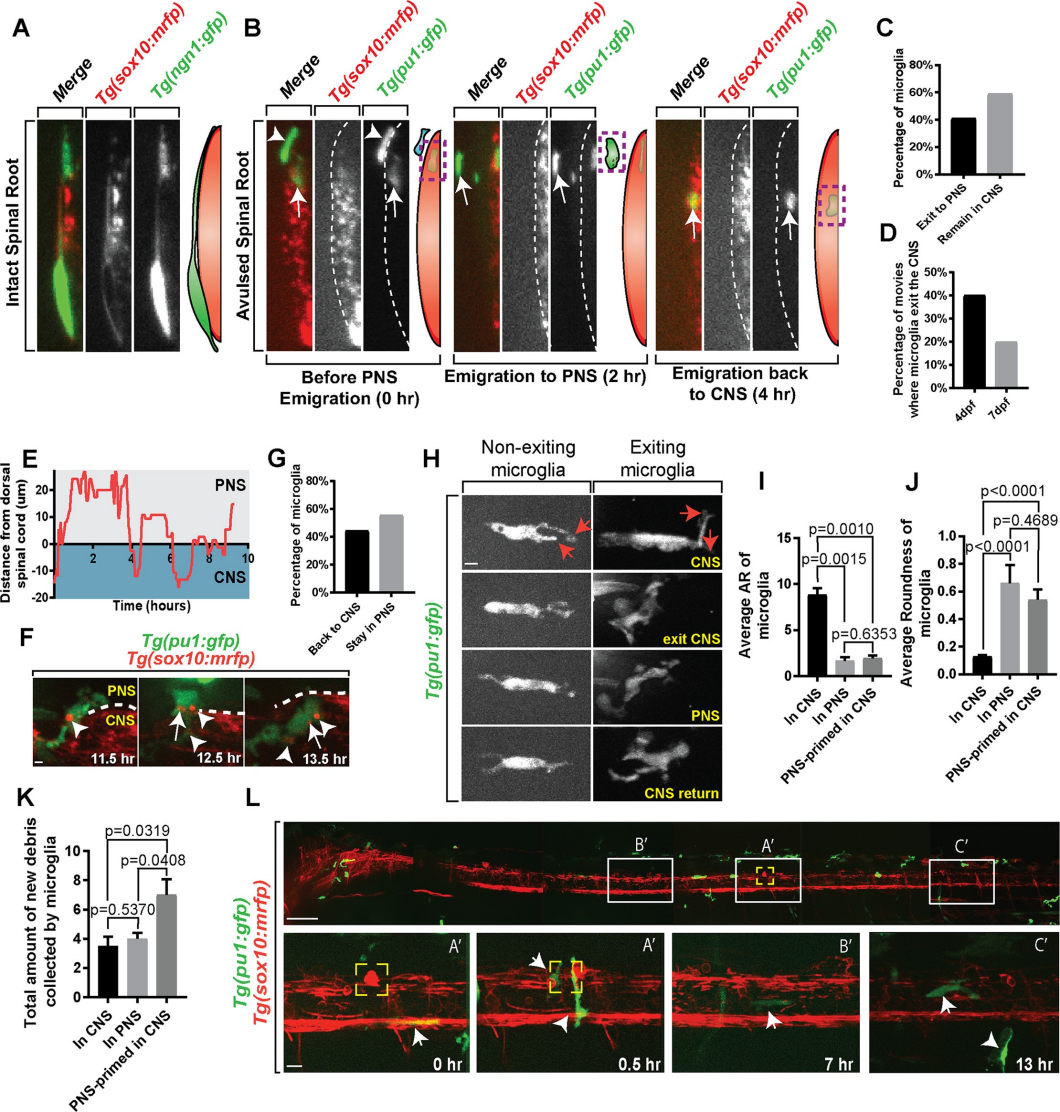
Microglia emigrate to the PNS and changes states following avulsion. (A) Orthogonal rotation view of *Tg(ngn1*:*gfp);Tg(sox10*:*mrfp)* animals showing the proximity of the intact spinal root to the spinal cord. (B) Orthogonal rotation view of *Tg(pu1*:*gfp);Tg(sox10*:*mrfp)* animals showing microglia before PNS emigration, during emigration, and post-emigration back to the CNS. Arrows and green diagram color indicate microglia. Arrowheads and blue diagram color indicate macrophages. Dashed line indicates spinal cord boundary. (C) Quantification of the percentage of microglia that exit the CNS compared with those that remain in the CNS. (D) Quantification of the percentage of movies in which microglia exit the CNS at 4 dpf and 7 dpf. (E) Representative quantification of distance and time a microglia spent inside and outside of the CNS; y-axis > 0 indicates cell’s presence in PNS; y-axis < 0 indicates cell’s presence in CNS. (F) Images from a 24-hour time-lapse movie starting at 4 dpf in *Tg(pu1*:*gfp);Tg(sox10*:*mrfp)* zebrafish showing microglia picking up debris while in the PNS and re-enter the CNS. Arrowheads indicate debris puncta while in CNS. Arrows indicate debris puncta while in the PNS. (G) Quantification of percentage of ectopic microglia that come back into CNS after leaving to the PNS compared with those that stay in PNS in a 24-hour imaging window. (H) Images from a 24-hour time-lapse movie starting at 4 dpf in *Tg(pu1*:*gfp);Tg(sox10*:*mrfp)* zebrafish comparing the morphology of exiting and nonexiting microglia. Red arrows indicate projections created by microglia. (I) Shape description quantification of the average aspect ratio of microglia in the CNS, in the PNS, and PNS-primed in the CNS (*p* = 0.0010; *p* = 0.0015; *p* = 0.6353). (J) Shape description quantification of the average roundness of microglia in the CNS, in the PNS, and PNS-primed in the CNS (*p* < 0.0001; *p* < 0.0001; *p* = 0.4689). (K) Quantification of the total amount of new debris collected by microglia (*p* = 0.0319; *p* = 0.5370; *p* = 0.0408). (L) Stitched zoning images from a 24-hour time-lapse movie starting at 4 dpf in *Tg(pu1*:*gfp);Tg(sox10*:*mrfp)* zebrafish showing the entire animal. White boxes coordinated with letter tags represented zones of the animal in which microglia traveled; larger images located below. Yellow box indicates injury site. Arrows indicate microglia. Arrowheads indicate macrophages. Scale bar equals 1 μm (F,H), 10 μm (bottom L), and 100 μm (top L). Statistics summarized in S1 Table. See S2 Data for raw data. CNS, central nervous system; dpf, days post fertilization; PNS, peripheral nervous system.

To determine the role of phagocytic cells in clearance of these domains, we tracked both cells in uninjured cases and following injury in lateral views in which we could distinguish the location of the spinal cord and the PNS (Fig 2A and 2B, S7A and S7B Fig). In these movies, we visualized GFP^+^ cells that originated in the CNS, migrate to the injury site, squeeze at the spinal cord boundary, and potentially migrate outside of its CNS domain (S7C and S7D Fig, S3 Movie). This hourglass-like morphology is typical of cells that must squeeze through a space-restricted area to leave the spinal cord [17,33]. We confirmed the ectopic migration of these cells by rotating our z-stack 90° in the movies; the distance of this migration also extended laterally beyond the normal range of the *gfap*^*+*^ spinal cord glial limitans (Fig 2A and 2B, S7E Fig). We measured these cells extended on average 14.7 μm beyond the glial limitans boundary; in comparision, the DRG was measured on average 4.8 μm outside the glial limitans (S7F Fig). We also visualized microglia in contact with the PNS-located DRG resident cells, supporting the hypothesis that microglia clearly exited the CNS (S7G–S7H Fig). We quantified this behavior in our 24-hour movies after injury and microglia were detected laterally outside of the mRFP region in 40% of the movies, they displayed an hourglass-like morphology, and in 3D reconstructions were located outside the normal region of the curvature of the glial limitans (Fig 2C, S7E Fig). In these emigration events, typically one to two microglia exited (S7I Fig). This is in contrast to noninjured spinal cords in which microglia never were present in these regions. On average, microglia emigrated 12.87 μm outside of the *sox10*^*+*^ CNS region following spinal root avulsion, remained there on average 4.13 hours, and displayed microglia-like morphology while positioned there (S7J and S7K Fig). In these emigration events, a continuous GFP^+^ process that remained in the CNS could not be detected, suggesting that the entirety of the microglia cell left the CNS. After emigration, individual microglia did return to the CNS (Fig 2B and 2E).

It is possible that this microglia emigration occurs from a massive disruption of the spinal cord boundary. However, despite disruption of the glial limitans in category III injury cases, microglia emigration was still observed in category I and II injury types with limited glial limitans disruption (S7L Fig). Also inconsistent with this idea, microglia emigration was preceeded by a squeezing of the microglia, suggesting it migrates through a space-restricted area (S7C and S7D Fig). If a general boundary disruption was present, oligodendrocytes and neurons would potentially ectopically exit as they do when boundary cap cells and/or Schwann cells are disrupted [15–18]. However, we also did not detect emigration of oligodendrocytes (S7L Fig). We next asked whether emigration was specific to root injuries by injuring the mixed PNS nerve (analogous to the sciatic nerve) and did not detect emigration (S8A–S8C Fig). Instead, and as previously reported, macrophages were the responding cell at mixed nerves [34](S8D and S8E Fig). Microglia also did not emigrate when we created a CNS-specific injury (S8C and S8E Fig). To rule out the possibility that this was specific to developmental properties at 4 dpf, we also observed microglia emigration to avulsions at 7 dpf (Fig 2D). Based on these data, we propose that microglia can exit the spinal cord, at least to the PNS-located spinal root and DRG, following OBPI-like injuries. Such an observation is in contrast to current textbook definitions of microglia.

### Emigrated microglia phagocytize PNS debris and re-enter the CNS

To gain further insight into this emigration, we tracked the trajectory of individual microglia after injury. Not only did microglia exit following injury, but this tracing analysis showed that individual microglia traverse the spinal cord boundary an average 6.25 times throughout their response to injury (Fig 2E, S9A–S9I Fig). We next considered the hypothesis that, although microglia ectopically migrated, their phagocytic properties could be different between their resident domain and the ectopically located PNS domain. To test this, we took advantage of our imaging approach and tracked individual microglia after injury, then scored and tracked the individual debris concentrates within those microglia. In these movies, we could identify *pu1*^*+*^ cells that originated in the CNS contain mRFP^+^ debris (Fig 2F, S9J–S9M Fig). To test whether debris could be carried across the CNS boundary, we scored the appearance of individual mRFP^+^ clusters in the GFP^+^ cells in the CNS. During their emigration to the spinal root, on average two mRFP^+^ particles from the CNS were carried within the microglia to the PNS. And while in the PNS, an additional one mRFP^+^ debris appeared in the cell (Fig 2F, S9N Fig). This appearance of mRFP^+^ debris particles while in the PNS is unlikely from already present CNS debris particle fission because the area of individual particles increases from 2.05 μm^2^ to 2.83 μm^2^ while the microglia cell is migrating (S9O Fig). As the cells entered back into the CNS, mRFP^+^ debris particles that appeared while the cell was in the PNS continued to be present (Fig 2F). These data are consistent with the possibility that *pu1*^*+*^ microglia not only migrate to the PNS-located roots but also clear debris while there. Additionally, their entry back into the CNS with PNS debris introduces the CNS to PNS debris.

Given this movement of microglia, we next sought to determine where microglia and macrophages eventually reside following their injury response. To do this, we identified CNS versus PNS *pu1*^*+*^ cells before injury, created injuries, imaged those injury sites for 24 hours, and tracked the individual cells with tracking software. In this analysis of the microglia that exited to the PNS, 44.44% of them migrated back to the CNS, where they continued to reside until the end of the 24-hour imaging window (Fig 2G). This phenomonon is distinct from other CNS cells that have been shown to emigrate because microglia also re-enter [15,17]. In the other 55.56%, microglia continued to be present in the PNS at the injury site at the end of the 24-hour imaging window (Fig 2G). These PNS-located microglia did not leave the injury site during this time. Macrophages migrated into and out of the injury site, sometimes entering the CNS. However, at the end of the imaging window, macrophages were rarely seen in the CNS. The simplest explanation for this data is that microglia can leave the CNS to respond to avulsions and can return to CNS residency after clearing debris.

To dissect the consequence of this emigration, we tracked individual microglia that emigrated and then returned to the CNS. During this process, we measured numerous cellular properties that were previously described across species to indicate altered microglia [9]. We first tested whether the morphology of single microglia changed as they progressed through their emigration and re-entry (Fig 2H, S10A–S10D Fig). To determine morphological changes, we measured four shape descriptors. Microglia showed a signficant shift in aspect ratio (an average measure of 8.57) and cell roundness (an average measure of 0.21) as cells were leaving the CNS (Fig 2I and 2J, S10B and S10C Fig). These differences remained while the cell returned to the CNS. To address whether this was a result of their location at the injury as apposed to emigration, we compared emigrating microglia to microglia that responded to and actively cleared debris at the injury site but never exited the CNS (S10E–S10H Fig). Again, this analysis showed that aspect ratio and roundness were different from cells that emigrated compared to nonexiting microglia. We could not detect any differences in the cells before emigration (Fig 2H–2J, S10I and S10J Fig), inconsistent with the hypothesis that emigrating microglia are distinct, at least morphologically, before emigration.

### PNS-experienced microglia return in an altered state

To further dissect whether microglia are altered, we tested whether emigrated microglia were physiologically changed by scoring their phagocytic activity. Using movies, we could score the number of new debris particles within individual microglia. We scored that individual microglia before their emigration increase the number of new mRFP^+^ particles once they return to the CNS (Fig 2K, S10K Fig). As a third indicator of altered microglia, we also scored the number of secondary projections that are used in the phagocytic process [9,23] (S10L Fig). Consistent with the conclusion that emigrated microglia return in an altered state, they increase their secondary projections while in the PNS and remain elevated as they re-enter (S10L Fig). Emigrated microglia also were distinct from microglia that responded to injuries of CNS tissue only (S10M Fig). Together, these data are consistent with the idea that emigration itself could induce a unique microglial state.

Because CNS-resident *pu1*^*+*^ cells migrated out of the spinal cord but then returned to the CNS, we next asked whether those ectopically migrated and altered cells moved to distal areas of the CNS following their emigration. To do this, we created injuries in animals, tiled the animal from the brain to tail with confocal positions, and time-lapse imaged each of these positions for 24 hours. With this whole spinal cord analysis, we could visualize CNS-derived *pu1*^*+*^ cells migrate to the injury, squeeze into the PNS, relocate to the CNS through the spinal cord, and then migrate anteriorly toward the brain and then caudally to the tail, surveying 743.56 μm (56.61%) of the spinal cord on average (Fig 2L, S11A–S11E Fig, S4 Movie). These movies demonstrated PNS-primed microglia that carried debris from the injuries to the brain (S11A Fig). This tiling analysis also allowed us to see interactions between individual cells along the length of the animal. We commonly visualized emigrated microglia interacting with other CNS-resident microglia in the spinal cord (S11F–S11H Fig, S4 Movie). Based on these data, we conclude that not only can microglia migrate out of the CNS but they can re-enter the CNS as PNS-primed cells and migrate, in an altered state, to distal areas from the injury site. These migration sites include the brain.

### PNS-primed microglia display altered responses to secondary injuries

**Fig 3:**
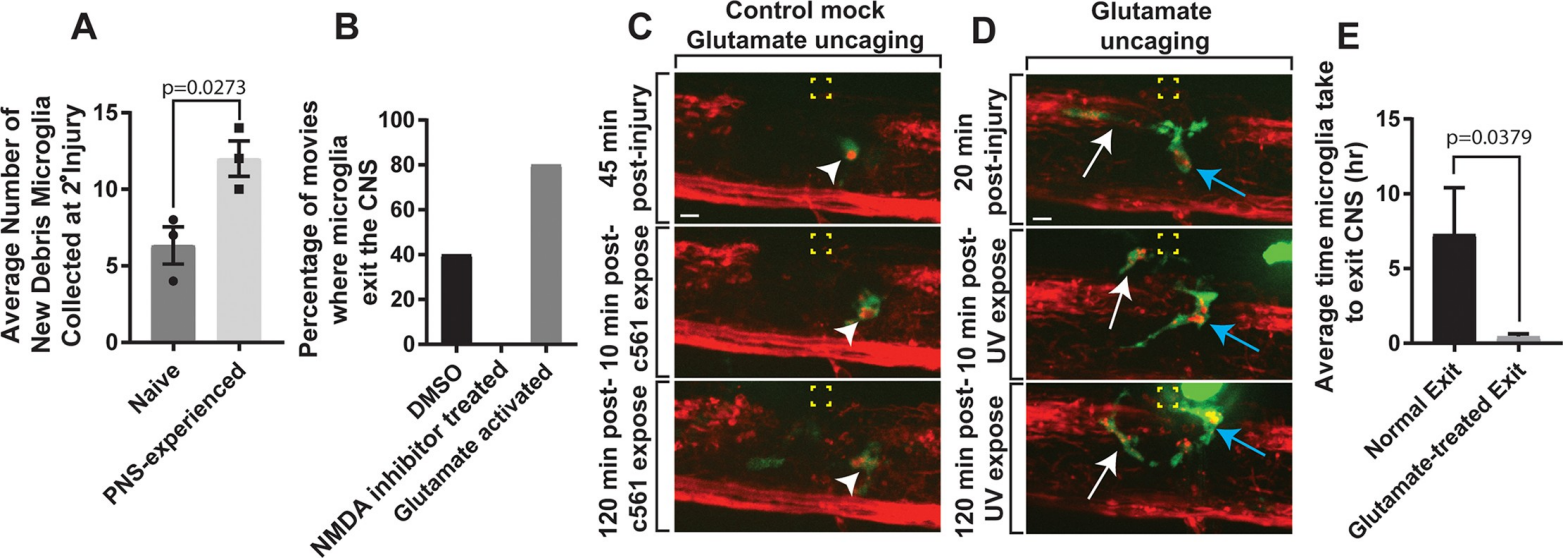
Glutamate-NMDA induces migration of microglia to the PNS. (A) Quantification of the average number of new debris from PNS-primed microglia collected at the secondary injury site compared to naïve CNS microglia (*p* = 0.0273). (B) Quantification of the percentage of movies in which microglia exit the CNS in DMSO, NMDA inhibitor treated, and glutamate uncaged in PNS cases. (C) Images from 24-hour time-lapse movies starting at 4 dpf in *Tg(pu1*:*gfp);Tg(sox10*:*mrfp)* MNI-L-glutamate treated zebrafish showing the response of microglia pre- and post-mock glutamate uncaging. Arrowheads denote microglia. Yellow box indicates uncaging site. (D) Images from 24-hour time-lapse movies starting at 4 dpf in *Tg(pu1*:*gfp);Tg(sox10*:*mrfp)* MNI-L-glutamate treated zebrafish showing the response and ectopic migration of microglia pre- and post-glutamate uncaging. White arrow denotes first microglia; blue arrow indicates a second microglia. Yellow box indicates uncaging site. (E) Quantification of the average time microglia took to exit the CNS post-glutamate uncaging (*p* = 0.0379). Scale bar equals 10 μm (C, D). Statistics summarized in S1 Table. See S3 Data for raw data. CNS, central nervous system; dpf, days post fertilization; MNI-L, 4-Methoxy-7-nitroindolinyl-caged-L-glutamate; NMDA, N-methyl-D-aspartate receptor; PNS, peripheral nervous system.

To begin to understand the potential functional consequence of microglia emigration, we created a primary avulsion injury in *Tg(pu1*:*gfp);Tg(sox10*:*mrfp)* animals at 4 dpf and observed emigration of microglia to the PNS. Then we created a distal secondary CNS-specific injury. Upon secondary injuries, we observed PNS-primed microglia immediately re-enter the CNS and migrate to the secondary injury site (S12A Fig). We quantified the amount of new debris PNS-primed microglia collected at the secondary injury site, which was greater than the amount of new debris naïve CNS microglia collected at the secondary injury site (Fig 3A). Naïve microglia were defined as never contacting the initial primary injury site. Additionally, PNS-primed microglia created more secondary projections at the secondary injury and spent more time there (S12B–S12D Fig). Together, these data demonstrate that PNS-primed microglia are more phagocytically active when they return to the CNS and could present an altered response to other injuries that occur after the avulsion.

### Inhibition of NMDA receptors prevents microglia emigration

To dissect the molecular mechanism of this emigration, we screened through small molecules that could disrupt emigration. In this, we identified that NMDA inhibitors, MK-801 and D-AP5 [30], disrupted the emigration of the microglia to the PNS (Fig 3B, S13A Fig). To dissect this mechanism further, we tracked individual microglia following injury in DMSO and NMDA inhibitor exposure. We first hypothesized that lack of NMDA signaling disrupted the response of the microglia to spinal sensory roots. However, by tracking individual *pu1*^*+*^ cells after NMDA inhibition, microglia and macrophages still responded to the injury, ruling out the possibility that blocking NMDA receptors prevented an initial injury response (S13B and S13C Fig). Instead, microglia responded to injury but did not emigrate (Fig 3B). This lack of emigration when NMDA signaling is disrupted perturbed the alteration of microglia at the injury that occurs as they emigrate (S14 Fig). Together, these data are consistent with the hypothesis that microglia exit of the CNS following injury is dependent on NMDA.

### Glutamate activation induces microglia emigration

To test this mechanism further, we asked whether glutamate, an activator of NMDA, could induce microglia emigration. We did this by soaking animals in caged glutamate [35], created brachial plexus injury (BPI)-like injuries, waited for a *pu1*^*+*^ cellular response to the injury, uncaged a 4 μm region in the PNS by exposing it to 405 nm laser, and then imaged for 2 hours after uncaging (Fig 3C and 3D, S5 Movie). As a control, soaking of caged glutamate did not alter the initial response time to the injury (Fig 3C). However, on average, the uncaging of glutamate induced exit of microglia to the PNS in 30 minutes. This was significnalty faster than controls: untreated injuries showed average exit in 7.29 hours and caged-glutamate soaked animals that were exposed to a 4 μm region in the PNS of 641 nm light did not exit before 2 hours (Fig 3E, S15A Fig). Uncaging glutamate induced emigration in the first 2 hours in 80% of injuries compared to 0% in mock-activated controls (S15A Fig). Microglia in both cases traveled the same distance, ruling out the possibility that these significant response times were caused from varying travel distances (S15B and S15C Fig). Consistent with their emigration, uncaging glutamate also caused morphological changes that occur in emigration states (S14 Fig, S15D–S15F Fig). To ask whether glutamate was sufficient without injury to induce emigration, glutamate was uncaged in the absence of injury (S15G and S15H Fig). Consistent with NMDA signaling inhibitors not reducing the initial injury response but specifically emigration, glutamate was not sufficient to induce emigration without injury. These data are consistent with the hypothesis that the mechanism of microglia emigration is NMDA- and glutamate-dependent.

### Interactions between microglia and macrophages control microglial emigration

**Fig 4:**
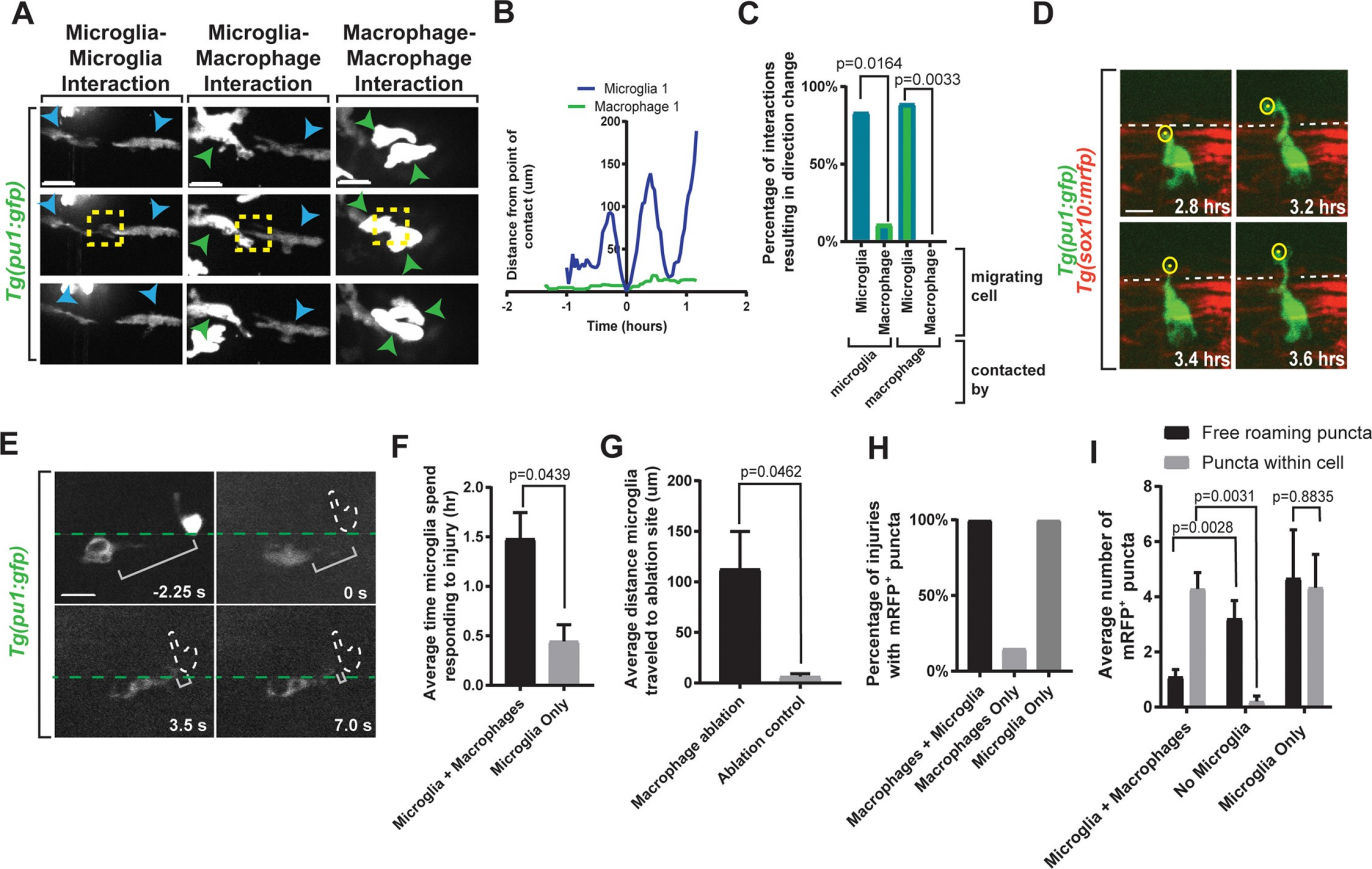
Heterotypic interactions between phagocytic cells alters emigration. (A) Images from 24-hour time-lapse movies starting at 4 dpf in *Tg(pu1*:*gfp);Tg(sox10*:*mrfp)* zebrafish showing points of cell–cell contact. Blue arrowheads indicate microglia. Green arrowheads indicate macrophages. Yellow box indicates contact. (B) Quantification of distance traveled pre- and post-contact between a microglia and macrophage. (C) Quantification of percentage of cells experiencing directional change post-contact with migrating cell (*p* = 0.0164; *p* = 0.0033). (D) Images from a 24-hour time-lapse movie starting at 4 dpf in *Tg(pu1*:*gfp);Tg(sox10*:*mrfp)* zebrafish showing microglia extending a cellular process into the periphery. Dashed line indicates *sox10*^*+*^ boundary. Yellow circle indicates tip of cellular projection. (E) Images from a time-lapse ablation window in *Tg(pu1*:*gfp)* zebrafish at 4 dpf showing successful ablation of macrophage and immediate microglial response. Green dashed line indicates spinal cord boundary. Bracket indicates distance between microglia and ablated macrophage. (F) Quantification of average time microglia spent responding to injury. (G) Quantification of average distance microglia traveled to the macrophage or control ablation site (*p* = 0.0462). (H) Quantification of percentage of injuries containing *sox10*^*+*^ puncta. (I) Quantification of number of free-roaming debris puncta compared to puncta localized within cells. Scale bar equals 10 μm (A, D, and E). Statistics summarized in S1 Table. See S4 Data for raw data. dpf, days post fertilization.

We hypothesized that a balance between NMDA induction and an emigration restriction mechanism determined emigration efficiency. Domain-specific cells can be restrictive to cells outside and inside of their domain [16,17,25,36–38]. Given that both macrophages and microglia responded to injury, we hypothesized that the presence of specific cells at the injury site, like macrophages, could prevent microglia from performing their full debris-clearing potential. We tested this mechanism in our imaging set-up by initially scoring interactions between the distinct *pu1*^*+*^ cells (S6 Movie). We first quantified the number of times each cell type displayed homotypic versus heterotypic contact (Fig 4A and 4B, S16A–S16C Fig). Then, we asked whether those interactions induced directional changes. In this analysis, we identified that homotypic interactions between microglia induced migration of microglia 83.33% of the time and heterotypic interactions between microglia and macrophages induced migration of microglia away from the contacting cell 88.88% of the time (Fig 4C, S6, S7 and S8 Movies). Macrophages did not respond to either homotypic or heterotypic contact (Fig 4A and 4C, S16B–S16D Fig, S9 Movie). We could quantify this by measuring distance traveled over time and measured that microglia travel on average 157.25 μm after contact whereas macrophages travel 9.20 μm after contact (S16D Fig). We next hypothesized that these heterotypic and homotypic interactions could impact the ability of microglia to emigrate.

To first test this possibility, we visualized microglial migration in the absence of macrophages. To do this, we tailored the laser parameters to produce spinal root avulsions with limited peripheral injury. In these injuries, microglia responded, but macrophages did not (S17A Fig, S10 Movie). We then traced the behavior of individual microglia. In contrast to injuries with macrophage responses, microglia migrated to the PNS quicker to these injuries, remained in the PNS, and extended long cellular processes into the PNS that were not visualized when macrophages were present (Fig 4D, S17 Fig). These data are consistent with the cellular mechanism that macrophages inhibit microglia emigration. We tested this potential mechanism with a second approach by reducing macrophages with focal laser ablations. In these experiments, we created avulsions, then after *pu1*^*+*^ cells migrated to the injury, we laser ablated single macrophages in close proximity to microglia at the injury site (Fig 4E, S18A and S18B Fig, S11 Movie). Following these single macrophage ablations, microglia migrated into the empty space that was created from macrophage ablations within seconds (Fig 4F and 4G, S18C–S18E Fig). Although we cannot completely rule out that macrophage debris attracts microglia, as a control identical laser ablation exposure to adjacent nonmacrophage space did not provoke migration into that area. This is consistent with the idea that ablation does not itself—or debris it creates—induce this microglial response (Fig 4G, S19 Fig). The simplest explanation for this is that dynamic interactions between microglia and macrophages deter microglia from occupying the PNS-located injury site.

To test whether this cell–cell contact mechanism has functional implications on debris clearance, we again took advantage of the simplicity of the zebrafish system; during early developmental stages, macrophages are abundant, but microglia are limited in number in the spinal cord and approximately 5% of animals display no CNS *pu1*^*+*^ cells in the spinal cord at 4 dpf. In these rare microglia-less spinal cords, we injured the spinal root and scored the percentage of injuries containing the mRFP^+^ debris that concentrated into puncta by phagocytic cells (Fig 4H, S20 Fig). As mentioned above, mRFP^+^ particles appear as the microglia migrate to the injury (Fig 1F–1H, S6 Fig). In contrast to injuries in animals with microglia, mRFP^+^ debris puncta was not detected in injuries without microglia, suggesting that microglia are required for debris clearance (Fig 4I). In contrast, for injuries with no macrophages, debris clustered quicker at the injury site (Fig 4F–4G). And consistent with the role of microglia, this debris was present only within microglia. These results are consistent with the cellular mechanism that microglia are responsible for debris clearance following injury but their access is inhibited to the injury site by macrophages. This stymies their ability to arrive and efficiently clear debris in both the CNS and PNS following spinal root avulsion.

## Discussion

Here, we demonstrate that microglia, despite their textbook definition, can alter their domain-specific residency. In this model of OBPI, the emigrating microglia serve as the debris-clearing cell (S21 Fig). Their potential, however, is stymied by macrophages. Macrophages restrict access to the area via a contact-dependent mechanism; a principle that appears to be consistently utilized across neural cell type, yet still remains largely molecularly elusive [17,25,36,39–43]. Emigration is balanced by two distinct mechanisms—positive migration via NMDA dependence and negative via contact-dependent cellular restriction. Together, our results introduce the concept that microglia migration out of the CNS is not only possible in OBPI, but while there, they can clear PNS debris and return it to the CNS in an altered state—a consequence that could have longer-term implications in the health of the nervous system [13].

Microglia activation during embryonic development has been implicated in autism-like phenotypes [13,44]. This activation can be induced by pollutants that the pregnant mother is exposed to [13]. These results suggest that improper activation of microglia during early embryonic stages could have long-term consequences on neural circuits. Our results indicate that emigration of microglia to the PNS roots can induce similar morphological and phagocytic activity changes that occur in activated microglia [9]. In our injury paradigm, these microglia then re-enter the CNS and can migrate over 60% of the spinal cord; their migration capacity even extends into the brain despite the severity of the injury and the integrity of the glial limitans. PNS-primed microglia that re-enter the CNS can migrate to distal sites of CNS-specific secondary injury and are more phagocytically active in response to secondary injuries. Microglia have been implicated in neuropathic pain following PNS injury [14,45]. This neuropathic pain can be halted if microglia are depleted within the first days after the injury [14], indicating that the initial response and changes of microglia is imperative. In light of these studies, emigration could be essential in numerous postinjury pathologies.

In specific disease states, subsets of microglia are activated. These microglia are referred to as disease-associated microglia (DAMs). DAMs transition through a series of stages that include regulation of specific transcription components, changes in physical appearance, and alterations in phagocytic activity [46]. The emigrated microglia return to the CNS with altered morphology and increased phagocytosis. These results indicate that emigrated microglia may resemble DAMs. Although ideal, monitoring transcriptional changes that would confirm these similarities is difficult to perform in emigrated microglia, because first the cells must be identified in movies as emigrated and then single-cell transcriptional analysis would need to be performed. With the number of microglia that emigrate at 4 dpf per injury, such analysis is technically infeasable. Future studies that resolve these technical hurdles will be important. Such analysis will also reveal the distinct differences between microglia and macrophages at the injury site, such as, e.g., their Tyro3, Axl, and Mer (TAMs), which are a family of receptor tyrosine kinases [47].

Microglia and macrophages largely occupy distinct domains. However, during disease states like spinal cord injury, multiple scleroris, and metastatic cancer, these distinct cells can reside in the same domains [48–50]. Our time-lapse imaging data indicate that microglia are restricted to regions that macrophages occupy. The consequence of this in BPI-like injuries is that microglia cannot clear debris as quickly. It also may ensure abundant microglia are not free to migrate to the PNS where they can become altered to an emigration state. If microglia are resctricted by macrophages in other diseases states like those in this BPI model, it may perturb the normal survelliance functions of microglia. Recent reports demonstrate that macrophages can inhibit microglia in spinal injuries [20], providing complimentary data that the interaction of macrophages and microglia, which we visualize in our movies occur after contact, could have profound effects on pathological states. This could also have downstream consequences to nervous system circuits.

Together, our results indicate that microglia function could extend beyond their textbook-defined region. This adds to their already impressive and essential roles in nervous system homeostasis and disease pathogenesis.

## Materials and methods

### Ethics statement

All animal studies were approved by the University of Notre Dame Institutional Animal Care and Use Committee.

### Contact for reagent and resource sharing

Reagents are available upon request to Cody J. Smith (csmith67@nd.edu).

### Experimental model and subject details

Animal studies were approved by the University of Notre Dame IACUC as noted above. Zebrafish strains used for this study were AB, *Tg(ngn1*:*gfp)* [51], *Tg(sox10*:*mrfp)* [33], *Tg(pu1*:*gal4-uas*:*gfp)* [26], and *Tg(gfap*:*gfp)* [52]. Pairwise matings were used to produce embryos and raised at 28°C in egg water in constant darkness. Animals were staged by hours post fertilization (hpf) or dpf [53]. Embryos of either sex were used for all experiments, and stable, germline transgenic lines were used.

### Method details

#### In vivo imaging

Animals were anesthetized with 3-amino-benzoic acid ester (Tricaine), covered in 0.8% low-melting point agarose, and mounted on their right side in glass-bottomed 35 mm Petri dishes [25]. Images were acquired on a spinning disk confocal microscope custom build by 3i technology that contains Zeiss Axio Observer Z1 Advanced Mariana Microscope; X-cite 120LED White Light LED System; filter cubes for GFP and mRFP; a motorized X,Y stage; a piezo Z stage; 20× Air (0.50 NA), 63× (1.15 NA), 40× (1.1 NA) objectives; CSU-W1 T2 Spinning Disk Confocal Head (50 μM) with 1× camera adapter and an iXon3 1Kx1K EMCCD camera; dichroic mirrors for 446, 515, 561, 405, 488, 561, and 640 excitation; laser stack with 405 nm, 445 nm, 488 nm, 561 nm, and 637 nm with laserstack FiberSwitcher; photomanipulation from vector high-speed point scanner ablations at diffraction limited capacity; and Ablate Photoablation System (532 nm pulsed laser; pulse energy 60 J at 200 Hz). Images in time-lapse microscopy were collected every 1 to 5 minutes for 2 to 24 hours depending on the experiment. Adobe Illustrator (https://www.adobe.com/products/illustrator.html, San Jose, CA) and ImageJ (https://imagej.nih.gov/ij/download.html, Bethesda, MD) were used to process images. Only brightness and contrast were enhanced for the presented images.

#### Laser-induced spinal avulsion

*Tg(sox10*:*mrfp);Tg(pu1*:*gfp)* 4-dpf animals were anesthetized using 0.02% 3-aminobenzoic acid ester (Tricaine) in egg water. Fish were then mounted in 0.8% low-melting point agarose solution, arranged laterally on a 10 mm glass coverslip-bottom Petri dish, and placed on the microscope anterior to posterior. Avulsion region of interest was mid-animal and selected upon location of DRG outside the spinal cord. Specific site of laser-induced injury was determined by tracing afferent projections from the DRG into the spinal cord using the z-plane. Projections with greater fluorescence and successful tracing were selected as the site of injury. This area was marked and brought into a focused ablation window. Upon focusing the targeted afferent projection, we moved 1 μm laterally out of the z-plane and double-clicked on the projection using a 4 μm cursor tool. All laser parameters used are specific to our confocal microscope. Specific parameters include Laser Power (2), Raster Block Size (1), Double-Click Rectangle Size (8), and Double-Click Repetitions (4).

#### Avulsion injury categorization

A catalog of all injuries performed was created and analyzed to create three separate categories of injury. Category I injuries are less severe, involving cutting the central projection and little to no surrounding damage. Category II injuries are more severe than involved severance of the central projection and some surrounding damage. Category III injuries are the most severe, involving severance of the central projection, visible damage to the spinal cord, and free-roaming debris. ImageJ was used to measure the length and width of the injury site created. ImageJ was also used to measure the average fluorescence of the injury site before and after the avulsion. These measurements were used to create a range of length, width, and fluorescence to place each avulsion case into a category. These ranges are listed in S2 Table. An avulsion case must meet at least two of the three catalog factors to qualify for a specific injury category.

#### Immunohistochemistry

The primary antibodies used in the assessment of boundary disruption were anti-Sox10 [54] (1:5,000, rabbit, Kucenas Lab) and anti-GFAP [52] (1:600, mouse, Dako). The secondary antibodies used were Alexa Fluor 647 goat antimouse (1:600, Invitrogen) and Alexa Fluor 560 goat antirabbit (1:600; Invitrogen). The primary antibody used in the confirmation of microglia in the brain and spinal cord compared to macrophages was anti-4C4 (1:50, mouse, Seiger, Becker, and Becker Labs) [27]. The secondary antibody used was Alexa Fluor 647 goat antimouse (1:600, Invitrogen). Larvae were fixed using 4% PFA in PBST (PBS, 0.1% Triton X-100) at 25°C for 3 hours. Fixed larvae were washed with PBST, DWT (dH_2_0, 0.1% Triton X-100), and acetone for 5 minutes each, then were incubated in −30°C acetone for 10 minutes. Larvae were then washed three times with PBST for 5 minutes and incubated with 5% goat serum in PBST for 1 hour at 25°C. Then, the larvae were incubated with primary antibody solution for 1 hour at 25°C, then transferred to −4°C overnight. After three washes with PBST for 30 minutes each and a longer PBST wash for 1 hour, the larvae were incubated with secondary antibody solution for 1 hour at 25°C, then transferred to −4°C overnight. After three washes with PBST for 1 hour, larvae were stored in 50% glycerol in PBS at 4°C until imaging. Larvae were mounted, and confocal images were taken using the above protocol for in vivo imaging.

#### smFISH

The probe used in the confirmation of microglia in the spinal cord was *tmem119* (500 μL, Stellaris FISH). Larvae were fixed at 4 dpf using 1 mL fresh fix solution (10× PBS, dH_2_0, 4% methanol-free formaldehyde) for 30 minutes. Fixed larvae were then washed twice in 100% methanol for two minutes each and incubated in 100% methanol at (-25°C) overnight. After one wash of 50% methanol, 30% methanol, and 100% 1× PBS for 5 minutes each, the larvae were decapitated using a razor blade. The caudal portion of the animal was then permeabilized with 10 mg/mL proteinase K for 45 minutes at 25°C. Decapitated larvae were then washed twice in 1× PBST (PBS, 0.1% Tween-20) and incubated with 4% PFA at 25°C for 20 minutes. Fixed larvae were washed twice in 1× PBST for 5 minutes each. After 3 washes with 1× PBST for 10 minutes each, fixed larvae were incubated with 50% Stellaris Wash Buffer (20× SSC, deionized formamide, and nuclease-free H_2_O) in 1× PBST for 5 minutes. After two 30-minute washes in 100% Stellaris Wash Buffer at 37°C, fixed larvae were incubated in 1.2 μL probe mixture and 500 μL Stellaris Hyb Buffer (dextran sulfate, 20× SSC, deionized formamide, nuclease-free water) at 37°C overnight. After two washes with 37°C Stellaris Wash Buffer for 3 minutes each, larvae were washed 4 times for 15 minutes each in 37°C Stellaris Wash Buffer. Fixed larvae were washed an additional 3 times with 1X PBST at 25°C for 10 minutes each. Then, larvae were immediately mounted, and confocal images were taken using the above protocol for *in vivo* imaging.

#### CSF-1 inhibitors

The chemical reagents used for this study were GW2580 (ApexBio). Stock solutions of 1 μM, 10 μM, and 100 μM were stored at −20°C with concentrations of 1% in DMSO. All embryos were dechorionated at 24 hpf and incubated with 3 mL egg water until desired treatment time. Fish were treated at 3 dpf and 24 hours and 12 hours before imaging at 4 dpf. Control fish were incubated with 1% DMSO in egg water 24 hours and 12 hours before imaging.

#### Peripheral injury

*Tg(sox10*:*mrfp);Tg(pu1*:*gfp)* 4 dpf animals were anesthetized using 0.02% 3-aminobenzoic acid ester (Tricaine) in egg water. Fish were then mounted in 0.8% low-melting point agarose solution, arranged laterally on a 10 mm glass-coverslip–bottom Petri dish, and placed on the microscope anterior to posterior. Avulsion region of interest was midanimal and selected upon location of DRG outside the spinal cord. Specific site of laser-induced injury was determined by tracing peripheral projections from the DRG into periphery using the z-plane. Projections with greater fluorescence and successful tracing were selected as the site of injury. This area was marked and brought into a focused ablation window. Upon focusing the targeted afferent projection, we moved 1 μm laterally out of the z-plane and double-clicked on the projection using a 4 μm cursor tool. All laser parameters used are specific to our confocal microscope. Specific parameters include Laser Power (2), Raster Block Size (1), Double-Click Rectangle Size (8), and Double-Click Repetitions (4).

#### CNS-specific injuries

*Tg(sox10*:*mrfp);Tg(pu1*:*gfp)* 4 dpf animals were anesthetized using 0.02% 3-aminobenzoic acid ester (Tricaine) in egg water. Fish were then mounted in 0.8% low-melting point agarose solution, arranged laterally on a 10 mm glass-coverslip–bottom Petri dish, and placed on the microscope anterior to posterior. Avulsion region of interest was midanimal and selected upon location of DRG outside the spinal cord. Specific site of laser-induced injury was determined by selecting a region inside the CNS where several oligodendroctyes, oligodendrocyte progenitor cells, and *sox10*^*+*^ axons were present between two DRGs using the z-plane. This area was marked and brought into a focused ablation window. Upon focusing the targeted deep CNS region, we moved 1 μm laterally out of the z-plane and double-clicked on a *sox10*^*+*^ region using a 4 μm cursor tool. All laser parameters used are specific to our confocal microscope. Specific parameters include Laser Power (1), Raster Block Size (1), Double-Click Rectangle Size (4), and Double-Click Repetitions (4).

#### Secondary injuries

*Tg(sox10*:*mrfp);Tg(pu1*:*gfp)* 4 dpf animals were anesthetized using 0.02% 3-aminobenzoic acid ester (Tricaine) in egg water. Fish were then mounted in 0.8% low-melting point agarose solution, arranged laterally on a 10 mm glass-coverslip–bottom Petri dish, and placed on the microscope anterior to posterior. First, a primary region of interest was selected, and an initial primary avulsion was created following the procedure as previously described in the laser-induced spinal avulsion section, using all the same laser parameters. We observed microglia responding to injury by checking the time lapse of the injury site every 15 minutes. After a microglia emigration event occurred, we immediately moved to a new posterior imaging region that did not overlap with the primary injury site. Then, we created a secondary injury in the CNS caudal to the primary injury site following the laser-avulsion procedure previously described in the CNS-specific injury section using the same laser parameters. We observed PNS-primed microglia response to secondary injury by imaging both the primary and secondary injury sites every 5 minutes for 24 hours.

#### NMDA inhibitors

The chemical reagents used for this study were MK-801 and D-AP5 (Tocris). Stock solutions of 25 mM MK-801 and 50 mM D-AP5 were stored at −20°C with concentrations of 1% DMSO [30]. Working solutions were diluted to 50 μM for MK-801 treatments and 30 μM for D-AP5 treatments. All embryos were dechorionated at 24 hpf and incubated with 3 mL egg water until desired treatment time. Fish were treated at 4 dpf, 2 hours before imaging. Control fish were incubated with 1% DMSO in egg water 2 hours before imaging.

#### NMDA injury

*Tg(sox10*:*mrfp);Tg(pu1*:*gfp)* 4 dpf MK-801 and D-AP5 treated animals were anesthetized using 0.02% 3-aminobenzoic acid ester (Tricaine) in egg water. Fish were then mounted in 0.8% low-melting point agarose solution, arranged laterally on a 10 mm glass-coverslip–bottom Petri dish, and placed on the microscope anterior to posterior. Microglia were identified in the spinal cord by taking confocal z-stack images and rotating each image 90 degrees to verify the cell was inside the CNS. An avulsion of the region of interest was performed following the spinal root avulsion protocol above to elicit microglial response to the injury site. Migration of microglia to the injury post-avulsion was imaged every 5 minutes for 24 hours.

#### Glutamate treatment

The chemical reagents used for this study were MNI-caged-L-glutamate (Tocris). Stock solutions of 50 mM were stored at −20°C with concentrations of 1% DMSO. Working solutions were diluted to 10 μM. All embryos were dechorionated at 24 hpf and incubated with 3 mL egg water until desired treatment time [35]. Fish were treated at 4 dpf with 10 μM caged glutamate 1 hour before imaging. Control fish were incubated with 1% DMSO in egg water 1 hour before imaging.

#### Glutamate uncaging

*Tg(sox10*:*mrfp);Tg(pu1*:*gfp)* 4 dpf glutamate-treated animals were anesthetized using 0.02% 3-aminobenzoic acid ester (Tricaine) in egg water. Fish were then mounted in 0.8% low-melting point agarose solution, arranged laterally on a 10 mm glass-coverslip–bottom Petri dish, and placed on the microscope anterior to posterior. Microglia were identified in the spinal cord by taking confocal z-stack images and rotating each image 90 degrees to verify the cell was inside the CNS. An avulsion of the region of interest was performed following the spinal root avulsion protocol above to elicit microglial response to the injury site. Migration of microglia to the injury post-avulsion was imaged and directly monitored every 5 minutes by placing markers on the screen during the imaging cycle to track movement. Upon arrival to the injury site, the imaging cycle was stopped to begin the uncaging process. Glutamate uncaging was carried out using UV light on the fluorescent region of the confocal. The specific site of UV exposure was in the same area as the intial avulsion. The region of interest was exposed to the laser line 405 nm for 5 ms to uncage glutamate. Immediately following UV exposure, a time lapse was initiatied to take an image every 2 minutes for the desired imaging window. UV exposure used a 4 μm cursor tool. All laser parameters used are specific to our confocal microscope.

Glutamate uncaging controls (mock injury) were performed using anesthetized *Tg(sox10*:*mrfp);Tg(pu1*:*gfp)* 4 dpf animals treated with 10 μM L-glutamate 1 hour pre-avulsion. An avulsion was created in a specific region of interest, and microglial migration was monitored as described above. Upon arrival to the injury site, the imaging cycle was stopped, and “mock” uncaging was performed. Instead of using the 405 nm laser to uncage glutamate, the desired region of interest was exposed to the laser line 561 nm for 5 ms. Following exposure to v561, a time lapse was initiated to take an image every 2 minutes for the desired imaging window.

#### Single-cell ablations

*Tg(sox10*:*mrfp);Tg(pu1*:*gfp)* 4 dpf animals were anesthetized using 0.02% 3-aminobenzoic acid ester (Tricaine) in egg water. Fish were then mounted in 0.8% low-melting point agarose solution, arranged laterally on a 10 mm glass-coverslip–bottom Petri dish, and placed on the microscope anterior to posterior. Confocal z-stack images of *Tg(sox10*:*mrfp);Tg(pu1*:*gfp)* 4 dpf animals were taken preinjury. Injuries were then created following the laser-induced injury methods described above to induce both macrophages and microglia to the injury site. Time-lapse images of *pu1*^*+*^ cellular response to injury was monitored every minute for 2 hours. We checked for the presence of *pu1*^*+*^ cells at site of injury every 5 minutes. After 30 to 60 minutes, *pu1*^*+*^ cells arrived to the injury site, and the time lapse was stopped. Macrophages and microglia were confirmed by preimage z-stacks and post–spinal-injury time lapse movies and tracing cells back to their original domains. A confirmed macrophage was chosen and brought into a focused ablation window. Upon focusing the targeted cell, we double-clicked on the center of the cell body using a 4 μm cursor tool to fire the ablation laser. All laser parameters used are specific to our confocal microscope. Specific parameters include Laser Power (2), Raster Block Size (4), Double-Click Rectangle Size (8), and Double-Click Repetitions (4).

### Quantification and statistical analysis

To generate composite z-images for the cell, 3i Slidebook software (Denver, CO) was used. Individual z-images were sequentially observed to confirm composite accuracy. All graphically presented data represent the mean of the analyzed data unless otherwise noted. Cell tracking was performed using the MTrackJ plugin for ImageJ (https://imagescience.org/meijering/software/mtrackj/, Bethesda, MD). GraphPad Prism software (San Diego, CA) was used to determine statistical analysis. Full detail of the statistical values can be found in S1 Table.

#### Quantification of emigration

To track the ectopic migration of microglia, single cells were tracked using the MTrackJ plugin of ImageJ. *pu1*^*+*^ cells were imaged for 2 hours premigration to ensure the normal domain residency. After injury, cells were tracked from inside the spinal cord to the injury site. Three specific criteria were used to score the ectopic migration: (1) Cells migrated dorsolateral outside of the *sox10*:*mrfp*^*+*^ range in the spinal cord specifically at the injury site. (2) Cells displayed an hourglass-like morphology that is typical of cells that ectopically migrate from the spinal cord. (3) In visualization of 3D reconstruction of the injury site, the *pu1*^*+*^ cells appeared outside of the *sox10*^*+*^ boundary and curvature of the glial limitans. All migrations that did not definetly meet these criteria were conservatively scored as nonectopic migration. To confirm the ectopic exit, we combined transgenes to label the edge of the spinal cord. As a limit of transgenes and the transient nature of this exiting phenomenon, we combined analysis of *Tg(sox10*:*mrfp); Tg(gfap*:*gfp)* and *Tg(sox10*:*mrfp); Tg(pu1*:*gfp)*. We rotated these images 90 degrees to reveal a cross-section of the spinal cord. We then merged the two *Tg(sox10*:*mrfp)* channels to align the spinal cord, then merged the two merged images of *Tg(sox10*:*mrfp); Tg(gfap*:*gfp)* versus *Tg(sox10*:*mrfp); Tg(pu1*:*gfp)* to confirm the *pu1*^*+*^ cells that originated from the CNS were clearly outside the normal *gfap*^*+*^ spinal cord glial limitans.

#### Shape descriptors

The four shape descriptors used to analyze microglia morphology (circularity, aspect ratio, roundness, and solidity) were measured using ImageJ. ImageJ utilizes the following formulas to calculate each descriptor:

$$Circularity=\frac{4\pi \times area}{{\left(perimeter\right)}^{2}}$$

$$Aspect\;Ratio=\frac{major\;axis}{minor\;axis}$$

$$Roundness=\frac{4\times area}{\pi \times {\left(major\;axis\right)}^{2}}$$

$$Solidity=\frac{area}{convex\;area}$$

#### Quantification of *sox10*^*+*^ debris puncta

To track the presence of debris within microglia, single cells were tracked using the MTrackJ plugin of ImageJ. *pu1*^*+*^ cells imaged throughout the duration of time lapse imaging were analyzed before and after their arrival to laser-induced avulstion sites. The criteria for debris include that *sox10*:*mrfp*^*+*^ puncta were free roaming and were picked up by a *pu1*^*+*^ cell in the spinal cord or that *sox10*:*mrfp*^*+*^ puncta were already present inside a *pu1*^*+*^ cell and migrated with the direction of the *pu1*^*+*^ cell. Colocalization was confirmed by rotating the confocal images at varying time points 90 degrees. If the puncta were inside the *pu1*^*+*^ cells, they were considered debris. The area of the puncta were measured using ImageJ.

#### Quantification of migration

To track the individual paths of macrophage and microglia migration, the MTrackJ plugin on ImageJ was used. The center of each cell body was traced over time, and quantitative data were collected by ImageJ. Resulting x and y coordinates of each cell were overlayed to create migration plots. All distance and time points were calculated by the MTrackJ software and further quantified using Microsoft Excel (Redmond, WA).

#### Quantification of directional changes

The MTrackJ plugin on ImageJ was used for tracing. Initial direction change paths were traced by tracking the location of the center of the cell body. Contact-related directional changes were traced by tracking each cell projection that came into contact with another cell projection. All numerical data were collected by the MTrackJ plugin, and the resulting x and y coordinates of each cell track were used to perform all quantitative analyses.

## Supporting information

S1 Table Summary of statistical analysis. Values of total number of cells and animals scored for each figure panel and statistical test used to determine significance.
(XLSX)

S2 Table Criterita for the characterization of injuries. Value criteria of injuries created. These criteria determine the overall category of the injury. An injury must meet at least two criteria to be classified as a specific category.
(XLSX)

S1 Fig Laser induced avulsion in different CNS and/or PNS domains. (A) Quantification of decrease in fluorescent signal along the central sensory DRG projection post-injury in *Tg(ngn1*:*gfp)* zebrafish at 4 dpf. Gray box indicates lesion site. (B) Schematic of laser-induced avulsion model involving central and peripheral injury. (C) Quantification of decrease in fluorescent signal along the peripheral sensory DRG projection post-injury in *Tg(ngn1*:*gfp)* zebrafish at 4 dpf. Gray box indicates lesion site. (D) Confocal z-projection of *Tg(ngn1*:*gfp)* zebrafish at 4 dpf showing peripheral injury post-injury. Note that the lesion is specific to the laser exposure site. Red boxes indicate injury site. Scale bar equals 10 μm (D). See S5 Data for raw data. CNS, central nervous system; dpf, days post fertilization; DRG, dorsal root ganglia; PNS, peripheral nervous system.
(TIF)

S2 Fig Categorization of injuries. (A) Confocal z-projections of *Tg(sox10*:*mrfp)* zebrafish 4 dpf pre- and post-ablation to create category I, II, or III injuries. Qualifications for injury categorization listed in S2 Table. (B) Representative quantification of the intensity over background pre- and post-category I injury. (C) Representative quantification of the intensity over background pre- and post-category II injury. (D) Representative quantification of the intensity over background pre- and post-category III injury. Also, see S2 Table for specific categorical injury parameters. Scale bar equals 10 μm (A). See S6 Data for raw data. dpf, days post fertilization.
(TIF)

S3 Fig Boundary description of the glial limitans during avulsion. (A) Confocal z-stack images taken at 4 dpf in *Tg(ngn1*:*gfp)* zebrafish stained with *anti-GFAP* and *anti-*Sox10 antibodies comparing the integrity of spinal cord boundary across all injury categories. (B) Orthogonal rotation view of *Tg(pu1*:*gfp);Tg(sox10*:*mrfp)* animals stained with anti-GFAP showing the GFAP^+^ boundary of the spinal cord after each injury category. Red dashed line indicates absence of GFAP. (C–E) Quantification of the average fluorescence of GFAP present in control vs category I (C), II (D), and III (E) injuries. Red box equals *gfap* absence. Scale bar equals 10 μm (A). See S7 Data for raw data. dpf, days post fertilization; GFAP, glial fibrillary acidic protein.
(TIF)

S4 Fig Identification of microglia. (A) Rotated orthogonal view image from a 24-hour time-lapse movie using *Tg(pu1*:*gfp);Tg(sox10*:*mrfp)* zebrafish at 4 dpf showing microglia inside the spinal cord and a macrophage outside the spinal cord. Dotted lines indicate spinal cord boundary. (B) Graphical representation of 3D image described in (A). (C) Quantification of average number of *pu1*^*+*^ cells present per 300 μm region post-treatment with various GW2580 drug concentrations. (D) Quantification of average number of microglia present in the animal upon GW2580 treatments. (E) Quantification of the percentage of animals with no microglia in the spinal cord upon treatment with GW2580. (F) Confocal z-stack images taken from a *Tg(pu1*:*gfp);Tg(sox10*:*mrfp)* animal stained with *smFISH tmem119*. (G) Images from a 30-minute time-lapse movie starting at 4 dpf in *Tg(pu1*:*gfp);Tg(sox10*:*mrfp)* zebrafish showing that microglia are not associated with vasculature. Arrows indicate microglia. Arrowheads indicate macrophages in vasculature. Dashed lines indicate blood vessels. Scale bar equals 10 μm (F, G). See S8 Data for raw data. dpf, days post fertilization.
(TIF)

S5 Fig Microglia response time. (A) Images from a 24-hour time-lapse movie starting at 4 dpf in *Tg(pu1*:*gfp);Tg(sox10*:*mrfp)* zebrafish showing microglia responding to injury. (B) Quantification of the average velocity of injury response between microglia and macrophages. (C) Quantification of the average number of microglia or macrophages responding to each injury category. (D) Quantification of the percentage of macrophages and microglia the respond to each injury category. (E) Representative migration plot of three macrophages (grey) and one microglia (blue) displaying response of both cells to injury site. (F) Quantification of individual distances microglia and macrophages traveled from their original location to the injury site. (G) Quantification of percentage of phagocytic cells first to arrive at injury site. Scale bar equals 10 μm (A). See S9 Data for raw data. dpf, days post fertilization.
(TIF)

S6 Fig Debris-clearing capacity of microglia and macrophages. (A) Quantification of individual vacuoles per microglia and macrophage. (B) Quantification of individual vacuoles per macrophage before and during injury response. (C) Quantification of average time microglia spend responding to and clearing injury. (D) Quantification of amount of time macrophages spend responding to and clearing injury. See S10 Data for raw data.
(TIF)

S7 Fig Ectopic migration of microglia. (A) Images from a 24-hour time-lapse movie starting at 4 dpf in *Tg(pu1*:*gfp);Tg(sox10*:*mrfp)* zebrafish showing microglia exiting the CNS. (B) Orthogonal rotation view of *Tg(pu1*:*gfp);Tg(sox10*:*mrfp)* animals at 4 dpf with microglia present outside of the CNS. Arrows indicate microglia. Arrowheads indicate macrophages. Dashed line indicates spinal cord boundary. (C) Images from a 24-hour time-lapse movie starting at 4 dpf in *Tg(pu1*:*gfp);Tg(sox10*:*mrfp)* zebrafish showing microglia squeeze through the injury site. (D) Tracings of ectopically migrating microglia cells described in (C). (E) Overlayed confocal z-stack images from a *Tg(gfap*:*gfp);Tg(sox10*:*mrfp)* animal and a *Tg(pu1*:*gfp);Tg(sox10*:*mrfp)* animal showing the presence of microglia outside of the glial limitans. (F) Quantification of the distance from the outer z-plane edge of the DRG or microglia cell body to the edge of the spinal cord. (G) Confocal image and (H) traced schematic of an excerpt from a time-lapse movie following injury showing microglia in contact with PNS-located DRG cell bodies. (I) Quantification of the number of microglia per animal that are present in the imaging window that did or did not emigrate to the PNS. (J) Quantification of maximum distance ectopic microglia traveled outside of the *sox10*^*+*^ CNS. (K) Quantification of time microglia spent in PNS five hours post-ablation. (L) Quantification of the average number of oligodendrocytes and microglia that are present in the PNS at the site of avulsion. Scale bar equals 1μm (C) and 10 μm (A,G). See S11 Data for raw data. CNS, central nervous system; dpf, days post fertilization; DRG, dorsal root ganglia; PNS, peripheral nervous system.
(TIF)

S8 Fig Peripheral injuries do not elicit microglial response. (A) Representative migration plot of two macrophages (green) and one microglia (blue) displaying response of macrophages only to the site of distal peripheral injury. Red box indicates injury site. (B) Confocal z-projection of *Tg(nbt*:*dsred)* zebrafish 4 dpf pre- and post-distal peripheral ablation. (C) Quantification of the percentage of movies comparing microglia migration to sensory root avulsion, peripheral mixed nerve avulsion, and CNS-specific injury. (D) Quantification of the average number of microglia present in the imaging window compared with those that respond to peripheral mixed nerve injury or CNS-specific injury. (E) Quantification of the average number of macrophages present in the imaging window compared to those that respond to peripheral mixed nerve injury or CNS-specific injury. Scale bar equals 10 μm (B). See S12 Data for raw data. CNS, central nervous system; dpf, days post fertilization.
(TIF)

S9 Fig Domain specificity of microglia and macrophages. (A) Images from a 24-hour time-lapse movie starting at 4 dpf in *Tg(pu1*:*gfp);Tg(sox10*:*mrfp)* zebrafish showing microglia and macrophages in their typical domains pre-injury. Arrows indicate microglia. Arrowheads indicate macrophages. (B, D, F, H) Migration plots of individual microglia that have migrated to injury site and entered the PNS. (C, E, G, I) Quantification of distance and time a microglia cell spent inside and outside of the CNS. y-axis numbers > 0 indicate cell’s presence in PNS. y-axis numbers < 0 indicate cell’s presence in CNS. (J) Images from a 24-hour time-lapse movie starting at 4 dpf in *Tg(pu1*:*gfp);Tg(sox10*:*mrfp)* zebrafish showing pre-ectopic PNS migration of microglia. Arrows indicate microglia. Dotted line indicates dorsal edge of spinal cord. (K) Tracing of pre-ectopic microglia in (J). (L) Images from a 24-hour time-lapse movie starting at 4 dpf in *Tg(pu1*:*gfp);Tg(sox10*:*mrfp)* zebrafish showing migration of microglia to PNS. Arrows indicate microglia. Dotted line indicates dorsal edge of spinal cord. (M) Tracing of pre-ectopic microglia in (L). (N) Quantification of debris puncta within microglia collected before migration, during migration, and after migration. (O) Quantification of size of debris puncta within microglia collected before migration, during emigration, and after emigration. Scale bar equals 1 μm (J,L) and 10 μm (A). See S13 Data for raw data. CNS, central nervous system; dpf, days post fertilization; PNS, peripheral nervous system.
(TIF)

S10 Fig Differences between non-exiting and exiting microglia. (A–D) Shape descriptor quantification of the circularity (A), aspect ratio (B), roundness (C), and solidity (D) of microglia in the CNS, leaving the CNS, in PNS, re-entering the CNS, and PNS-primed in the CNS. (E–I) Quantification of the average circularity (E), aspect ratio (F), roundness (G), and solidity (H) of a PNS-primed microglia in the CNS versus a microglia that responded to injury but never left the CNS. (I) Shape descriptor quantification of the circularity of individual microglia in the CNS, in PNS, and PNS-primed in the CNS. (J) Shape descriptor quantification of the solidity of individual microglia in the CNS, in PNS, and PNS-primed in the CNS. (K) Quantification of the total amount of new debris a PNS-primed microglia collected compared to microglia that responded to injury but never left the CNS. (L) Quantification of the amount of primary and secondary projections created across different neuronal domains. (M) Quantification of the total amount of primary and secondary projections a PNS-primed microglia in the CNS versus a microglia that responded to injury but never left the CNS. See S14 Data for raw data. CNS, central nervous system; PNS, peripheral nervous system.
(TIF)

S11 Fig Microglia zoning tracks and replicates. (A) Stitched zoning images from a 24-hour time-lapse movie starting at 4 dpf in *Tg(pu1*:*gfp);Tg(sox10*:*mrfp)* zebrafish representing the entire spinal cord. White boxes coordinate with letter tags represent zones of the animal in which microglia traveled, larger images located below. Yellow box indicates injury site. Note that microglia traveled into brain region shown in (C). (B) Quantification of the percent of the animal surveyed by microglia. (C) Representative quantification of distance microglia traveled throughout the animal anteriorly and posteriorly. Red arrow indicates injury site, origin set to site of injury. (D) Quantification of the average distance each microglia travel throughout the CNS. (E) Quantification of the maximum distance each microglia travel throughout the CNS. (F) Stitched zoning images from a 24-hour time-lapse movie starting at 4 dpf in *Tg(pu1*:*gfp);Tg(sox10*:*mrfp)* zebrafish representing the entire animal and no microglia located within the CNS. Yellow boxes indicate injury site. (G) Images from a 24-hour time-lapse movie starting at 4 dpf in *Tg(pu1*:*gfp);Tg(sox10*:*mrfp)* zebrafish showing macrophage response to injury site. Yellow box indicates injury site. (H) Quantification of the total amount of heterotypic and homotypic cellular contacts. Scale bar equals 1 μm (bottom A), 10 μm (G), 100 μm (top A, F). See S15 Data for raw data. CNS, central nervous system; dpf, days post fertilization.
(TIF)

S12 Fig Secondary injuries and microglia response. (A) Images from a 24-hour time-lapse movie starting at 4 dpf in *Tg(pu1*:*gfp);Tg(sox10*:*mrfp)* zebrafish showing microglia arriving at the primary injury site, emigrating from the CNS, re-entering the CNS, and then migrating to the secondary injury site. Arrow indicates microglia. Yellow box indicates primary injury site. Blue box indicates secondary injury site. (B) Images from a 24-hour time-lapse movie starting at 4 dpf in *Tg(pu1*:*gfp);Tg(sox10*:*mrfp)* showing primary and secondary projections of PNS-experienced microglia at the secondary injury site. Green arrowheads indicate primary projections. Orange arrowheads indicate secondary projections. (C) Quantification of the average time microglia spent at the secondary injury site. (D) Quantification of the amount of primary and secondary projections PNS-experienced microglia created at the secondary injury site compared to naïve microglia. Scale bar equals 1 μm (B), 10 μm (A). See S16 Data for raw data. CNS, central nervous system; dpf, days post fertilization; PNS, peripheral nervous system.
(TIF)

S13 Fig NMDA treatment prevents ectopic migration. (A) Images from a 24-hour time-lapse movie starting at 4 dpf in *Tg(pu1*:*gfp);Tg(sox10*:*mrfp)* zebrafish comparing DMSO and NMDA inhibitor treated microglia response to the injury site. Arrowheads indicate microglia. (B) Quantification of the percentage of DMSO and NMDA-inhibitor treated animals that had microglia respond to the site of injury. (C) Quantification of the average number of microglia that are present and respond to the injury site. Scale bar equals 10 μm (A). See S17 Data for raw data. dpf, days post fertilization; NMDA, N-methyl-D-aspartate receptor.
(TIF)

S14 Fig Morphological differences between nonexiting and exiting microglia. (A-D) Shape descriptor quantification of the circularity (A), aspect ratio (B), roundness (C), and solidity (D) of DMSO compared to NMDA- and glutamate-treated individual microglia before exiting the CNS, in the PNS, and PNS-primed in the CNS. See S18 Data for raw data. CNS, central nervous system; NMDA, N-methyl-D-aspartate receptor; PNS, peripheral nervous system.
(TIF)

S15 Fig Glutamate induces exit of microglia after injury. (A) Quantification of the percentage of microglia that exit the CNS within 2 hours post-injury after no glutamate exposure, mock uncaging, and glutamate uncaging. (B) Quantification of the time it takes microglia to respond to injury post-normal injury versus post-glutamate uncaging. (C) Quantification of the distance microglia travel to the injury site post-normal injury versus post-glutamate uncaging. (D) Quantification of the average amount of debris individual microglia collect pre-CNS exit when treated with glutamate. (E) Quantification of the average area of the debris collected by individual microglia pre-CNS exit represented in (D). (F) Quantification of the average amount of debris individual microglia collect while in the PNS when treated with glutamate. (G) Images from a 24-hour time-lapse movie starting at 4 dpf in uninjured glutamate-treated *Tg(pu1*:*gfp);Tg(sox10*:*mrfp)* zebrafish showing no emigration of microglia to the PNS. Arrowheads indicate microglia. Yellow box indicates site of glutamate uncaging. (H) Quantification of the percentage of uninjured glutamate-treated animals with microglia present near the uncaging site compared to the percentage of microglia that exited the CNS following glutamate uncaging. Scale bar equals 10 μm (G). See S19 Data for raw data. CNS, central nervous system; dpf, days post fertilization; PNS, peripheral nervous system.
(TIF)

S16 Fig Heterotypic and homotypic interactions induce directional changes. (A) Images from a 24-hour time-lapse movie starting at 4 dpf in *Tg(pu1*:*gfp);Tg(sox10*:*mrfp)* zebrafish showing microglia experience directional changes. Arrowheads indicate microglia. Red circles indicate previous location of microglia. (B) Quantification of distance traveled pre- and post-contact between two microglia. (C) Quantificaion of distance traveled pre- and post-contact between two macrophages. (D) Quantification of average maximum distance traveled of each cell that experiences a directional change pre- and post-contact with a migrating cell (*p* = 0.0169, *p* = 0.0040). Scale bar equals 10 μm (A). Stats summarized in S1 Table. See S20 Data for raw data. dpf, days post fertilization.
(TIF)

S17 Fig Microglial response and ectopic migration post-injury. (A) Images from a 6-hour time-lapse movie starting at 4 dpf in *Tg(pu1*:*gfp);Tg(sox10*:*mrfp)* zebrafish showing the ectopic migration of microglia. Dotted line indicates dorsal edge of spinal cord. Yellow box indicates injury site. (B) Quantification of reaching events. y-axis numbers > 0 indicate projection presence in PNS. y-axis numbers < 0 indicate projection presence in CNS. (C) Quantification of microglial projection length in the CNS versus PNS. (D) 3D side view image from a 24-hour time-lapse movie starting at 4 dpf in *Tg(pu1*:*gfp);Tg(sox10*:*mrfp)* zebrafish showing microglial projection reaching into the PNS. (E) Graphical representation of events described in (D). Scale bar equals 1 μm (A). See S21 Data for raw data. CNS, central nervous system; dpf, days post fertilization; PNS, peripheral nervous system.
(TIF)

S18 Fig Microglial response post-macrophage ablation. (A) Images from a 2-hour time-lapse movie starting at 4 dpf in *Tg(pu1*:*gfp);Tg(sox10*:*mrfp)* zebrafish post-injury showing *pU1*^*+*^ cell responses to injury. (B) Images from a 2-hour time-lapse movie starting at 4 dpf in *Tg(pu1*:*gfp);Tg(sox10*:*mrfp)* zebrafish before and after single-cell macrophage ablation. Arrows indicate microglia. Arrowheads indicate macrophages. Dashed circles indicate ablated macrophages. (C) Images from 24-hour time-lapse movies starting at 4 dpf in *Tg(pu1*:*gfp);Tg(sox10*:*mrfp)* zebrafish post-macrophage ablation showing microglial response to ablation site. Arrows indicate microglia. Dashed circles indicate site of macrophage ablation. (D) Migration plot representing the migration of microglia directly to the site of macrophage ablation. (E) Quantification of the time and distance microglia traveled immediately following macrophage ablation. Red box indicates injury site. y-axis numbers > 0 indicates the PNS. y-axis numbers < 0 indicates the CNS. Scale bar equals 10 μm (A-C). See S22 Data for raw data. CNS, central nervous system; dpf, days post fertilization; PNS, peripheral nervous system.
(TIF)

S19 Fig Macrophage ablation controls. (A) Images from a 30-minute time-lapse movie at 4 dpf in *Tg(pu1*:*gfp);Tg(sox10*:*mrfp)* zebrafish showing the response of macrophages and microglia to the injury site. Arrows indicate microglia. Arrowheads indicate macrophages. Yellow box indicates injury site. (B) Images from a time-lapse ablation window in *Tg(pu1*:*gfp)* zebrafish at 4 dpf showing ablation control and no immediate microglial response. (C) Images from a 2-hour time-lapse movie at 4 dpf in *Tg(pu1*:*gfp);Tg(sox10*:*mrfp)* zebrafish showing no microglial response to the site of control ablation. Arrow indicates microglia. White circle indicates site of macrophage ablation. (D, F, H) Migration plots of microglia not responding to site of control ablations. Red box indicated control ablation site. (E, G, I) Quantification of distance microglia traveled pre- and post-control ablation over time. Red box indicates control ablation site. y-axis > 0 indicates cell’s presence in PNS. y-axis < 0 indicates cell’s presence in CNS. x-axis > 0 indicates after ablation. x-axis < 0 indicates before ablation. Scale bar equals 10 μm (A-C). See S23 Data for raw data. CNS, central nervous system; dpf, days post fertilization; PNS, peripheral nervous system.
(TIF)

S20 Fig Determination of *sox10^+^* debris puncta without microglia. Images from a 24-hour time-lapse movie starting at 4 dpf in *Tg(pu1*:*gfp);Tg(sox10*:*mrfp)* zebrafish post-injury showing macrophage response to injury in animals without microglia. Stills display how debris puncta were determined. Arrows indicate debris that is cleared. Arrowheads indicate debris that is not cleared. Dashed circles represent cleared debris. Scale bar equals 10 μm. dpf, days post fertilization.
(TIF)

S21 Fig Proposed model of injury response. (A) Model describing the observed nature of macrophages and microglia pre- and post-OBPI. (B) Model describing the glutamate-dependent emigration of microglia compared to unaltered microglia that remain in the CNS. (C) Model describing the factors necessary for the efficiency of microglia emigration after OBPI. CNS, central nervous system; OBPI, obstetrical brachial plexus injury.
(TIF)

S1 Movie Laser ablation of central sensory projection. Time-lapse movie starting at 4 dpf in *Tg(ngn1*:*gfp)* zebrafish showing the successful ablation of the central sensory projection. dpf, days post fertilization.
(MOV)

S2 Movie Phagocytic cell respond to injury. Excerpt from a 24-hour time-lapse movie starting at 4 dpf in *Tg(pu1*:*gfp);Tg(sox10*:*mrfp)* showing macrophage and microglia responding to injury. Yellow box indicates injury site. White triangle indicates microglia. Two untracked *pu1*^*+*^ cells at injury are macrophages. Note mRFP^+^ debris is only present in microglia. Frame rate equals 10 fps. dpf, days post fertilization; fps, frames per second; mRFP, membrane red fluorescent protein.
(MOV)

S3 Movie Ectopic migration of microglia. Excerpt from a 24-hour time-lapse movie starting at 4 dpf in *Tg(pu1*:*gfp);Tg(sox10*:*mrfp)* showing microglia exiting the spinal cord. Two untracked *pu1*^*+*^ cells at injury are macrophages. Yellow box indicates injury site. White triangle indicates microglia. Frame rate equals 10 fps. dpf, days post fertilization; fps, frames per second.
(MOV)

S4 Movie Microglia surveying entire zebrafish. 24-hour time-lapse movie starting at 4 dpf in *Tg(pu1*:*gfp);Tg(sox10*:*mrfp)* showing microglia surveying the whole animal anterior to posterior. White circle indicates microglia that responded to injury. Yellow box indicates injury site. Frame rate equals 10 fps. dpf, days post fertilization; fps, frames per second.
(MOV)

S5 Movie Glutamate uncaging facilitates emigration of microglia to PNS. 2-hour time-lapse movie starting immediately following a glutamate uncaging event in *Tg(pu1*:*gfp);Tg(sox10*:*mrfp)* animals. Following uncaging, a microglia immediately exits the spinal cord and enters the space where the uncaging occurred and continues to move throughout the PNS. Yellow box indicates site of glutamate uncaging. White triangle indicates microglia. Frame rate equals 1 fps. dpf, days post fertilization; fps, frames per second; PNS, peripheral nervous system.
(MOV)

S6 Movie Microglia struggle to respond to injury. Excerpt from a 24-hour time-lapse movie starting at 4 dpf in *Tg(pu1*:*gfp);Tg(sox10*:*mrfp)* showing microglia attempting to respond to injury. During injury response, the microglia experience several directional changes, producing a “zig zag” effect. Yellow box indicates injury site. White triangle indicates microglia. Frame rate equals 10 fps. dpf, days post fertilization; fps, frames per second.
(MOV)

S7 Movie Microglia homotypic interaction. Excerpt from a 24-hour time-lapse movie starting at 4 dpf in *Tg(pu1*:*gfp);Tg(sox10*:*mrfp)* showing an interaction between two microglia. After contact, both cells experience a directional change. Two blue triangles indicate microglia 1 (dark) and microglia 2 (light). Yellow box indicates region of homotypic contact. Frame rate equals 10 fps. dpf, days post fertilization; fps, frames per second.
(MOV)

S8 Movie Microglia and macrophage heterotypic interaction. Excerpt from a 24-hour time-lapse movie starting at 4 dpf in *Tg(pu1*:*gfp);Tg(sox10*:*mrfp)* showing an interaction between a microglia and a macrophage. After contact, the microglia shows a directional change while the macrophage remains stationary. Blue triangle indicates microglia. White triangle indicates macrophage. Yellow box indicates region of heterotypic contact. Frame rate equals 10 fps. dpf, days post fertilization; fps, frames per second.
(MOV)

S9 Movie Macrophage homotypic interaction. Excerpt from a 24-hour time-lapse movie starting at 4 dpf in *Tg(pu1*:*gfp);Tg(sox10*:*mrfp)* showing an interaction between two macrophages. After contact, neither cell displays a directional change. Two blue triangles indicate macrophage 1 (dark) and macrophage 2 (light). Yellow box indicates region of homotypic contact. Frame rate equals 10 fps. dpf, days post fertilization; fps, frames per second.
(MOV)

S10 Movie Microglia responds in absence of macrophages. Excerpt from a 24-hour time-lapse movie starting at 4 dpf in *Tg(pu1*:*gfp);Tg(sox10*:*mrfp)* showing microglia exiting the spinal cord. Over time the cell re-enters the spinal cord and creates projections that reach into the PNS. Yellow box indicates injury site. White triangle indicates microglia. White circle traces tip of microglial PNS projections. Frame rate equals 10 fps. dpf, days post fertilization; fps, frames per second; PNS, peripheral nervous system.
(MOV)

S11 Movie Microglial response to macrophage ablation. Time-lapse movie starting at 4 dpf in *Tg(pu1*:*gfp);Tg(sox10*:*mrfp)* showing the single-cell ablation of a macrophage and the microglial response immediately following ablation. Yellow triangle indicates microglia. Red box indicates area of macrophage ablation. Frame rate equals 10 fps. dpf, days post fertilization; fps, frames per second.
(MOV)

S1 Data Raw data represented in Fig 1. All raw data and statistical values represented in different spreadsheet tabs per figure for Fig 1E, 1G and 1H.
(XLSX)

S2 Data Raw data represented in Fig 2. All raw data and statistical values represented in different spreadsheet tabs per figure for Fig 2C, 2E, 2G, 2I and 2K.
(XLSX)

S3 Data Raw data represented in Fig 3. All raw data and statistical values represented in different spreadsheet tabs per figure for Fig 3A, 3B and 3E.
(XLSX)

S4 Data Raw data represented in Fig 4. All raw data and statistical values represented in different spreadsheet tabs per figure for Fig 4B, 4C, 4F and 4I.
(XLSX)

S5 Data Raw data represented in S1 Fig. All raw data and statistical values represented in different spreadsheet tabs per figure for S1A and S1C Fig.
(XLSX)

S6 Data Raw data represented in S2 Fig. All raw data and statistical values represented in different spreadsheet tabs per figure for S2B–S2D Fig.
(XLSX)

S7 Data Raw data represented in S3 Fig. All raw data and statistical values represented in different spreadsheet tabs per figure for S3C–S3E Fig.
(XLSX)

S8 Data Raw data represented in S4 Fig. All raw data and statistical values represented in different spreadsheet tabs per figure for S4C–S4E Fig.
(XLSX)

S9 Data Raw data represented in S5 Fig. All raw data and statistical values represented in different spreadsheet tabs per figure for S5B–S5G Fig.
(XLSX)

S10 Data Raw data represented in S6 Fig. All raw data and statistical values represented in different spreadsheet tabs per figure for S6A–S6D Fig.
(XLSX)

S11 Data Raw data represented in S7 Fig. All raw data and statistical values represented in different spreadsheet tabs per figure for S7F, S7I and S7L Fig.
(XLSX)

S12 Data Raw data represented in S8 Fig. All raw data and statistical values represented in different spreadsheet tabs per figure for S8A, S8C and S8E Fig.
(XLSX)

S13 Data Raw data represented in S9 Fig. All raw data and statistical values represented in different spreadsheet tabs per figure for S9B, S9I, S9N and S9O Fig.
(XLSX)

S14 Data Raw data represented in S10 Fig. All raw data and statistical values represented in different spreadsheet tabs per figure for S10A–S10M Fig.
(XLSX)

S15 Data Raw data represented in S11 Fig. All raw data and statistical values represented in different spreadsheet tabs per figure for S11B, S11E and S11H Fig.
(XLSX)

S16 Data Raw data represented in S12 Fig. All raw data and statistical values represented in different spreadsheet tabs per figure for S12C and S12D Fig.
(XLSX)

S17 Data Raw data represented in S13 Fig. All raw data and statistical values represented in different spreadsheet tabs per figure for S13B and S13C Fig.
(XLSX)

S18 Data Raw data represented in S14 Fig. All raw data and statistical values represented in different spreadsheet tabs per figure for S14A–S14D Fig.
(XLSX)

S19 Data Raw data represented in S15 Fig. All raw data and statistical values represented in different spreadsheet tabs per figure for S15A, S15F and S15H Fig.
(XLSX)

S20 Data Raw data represented in S16 Fig. All raw data and statistical values represented in different spreadsheet tabs per figure for S16B and S16D Fig.
(XLSX)

S21 Data Raw data represented in S17 Fig. All raw data and statistical values represented in different spreadsheet tabs per figure for S17B and S17C Fig.
(XLSX)

S22 Data Raw data represented in S18 Fig. All raw data and statistical values represented in different spreadsheet tabs per figure for S18D and S18E Fig.
(XLSX)

S23 Data Raw data represented in S19 Fig. All raw data and statistical values represented in different spreadsheet tabs per figure for S19D–S19I Fig.
(XLSX)

## Abbreviations

BPI: brachial plexus injury
CNS: central nervous system
CSF-1: colony stimulating factor-1
DAM: disease-associated microglia
dpf: days post fertilization
DRG: dorsal root ganglia
DWT: distilled water/tween
fps: frames per second
GFP: green fluorescent protein
GFAP: glial fibrillary acidic protein
DREZ: dorsal root entry zone
hpf: hours post fertilization
MNI: 4-Methoxy-7-nitroindolinyl-caged-L-glutamate
mRFP: membrane red fluorescent protein
NMDA: *N*-methyl-D-aspartate receptor
OBPI: obstetrical brachial plexus injury
PBS: phosphate-buffered saline
PBST: phosphate-buffered saline/tween
PFA: paraformaldehyde
PNS: peripheral nervous system
smFISH: small molecule fluorescent in situ hybridization
TAM: Tyro3, Axl, and Mer
