# Supplementary figures and images for: Microglia exit the CNS in spinal root avulsion
Source: PLoS Biol. 2019 Feb 22;17(2):e3000159. doi: 10.1371/journal.pbio.3000159 (PMC6402705; doi:10.1371/journal.pbio.3000159)

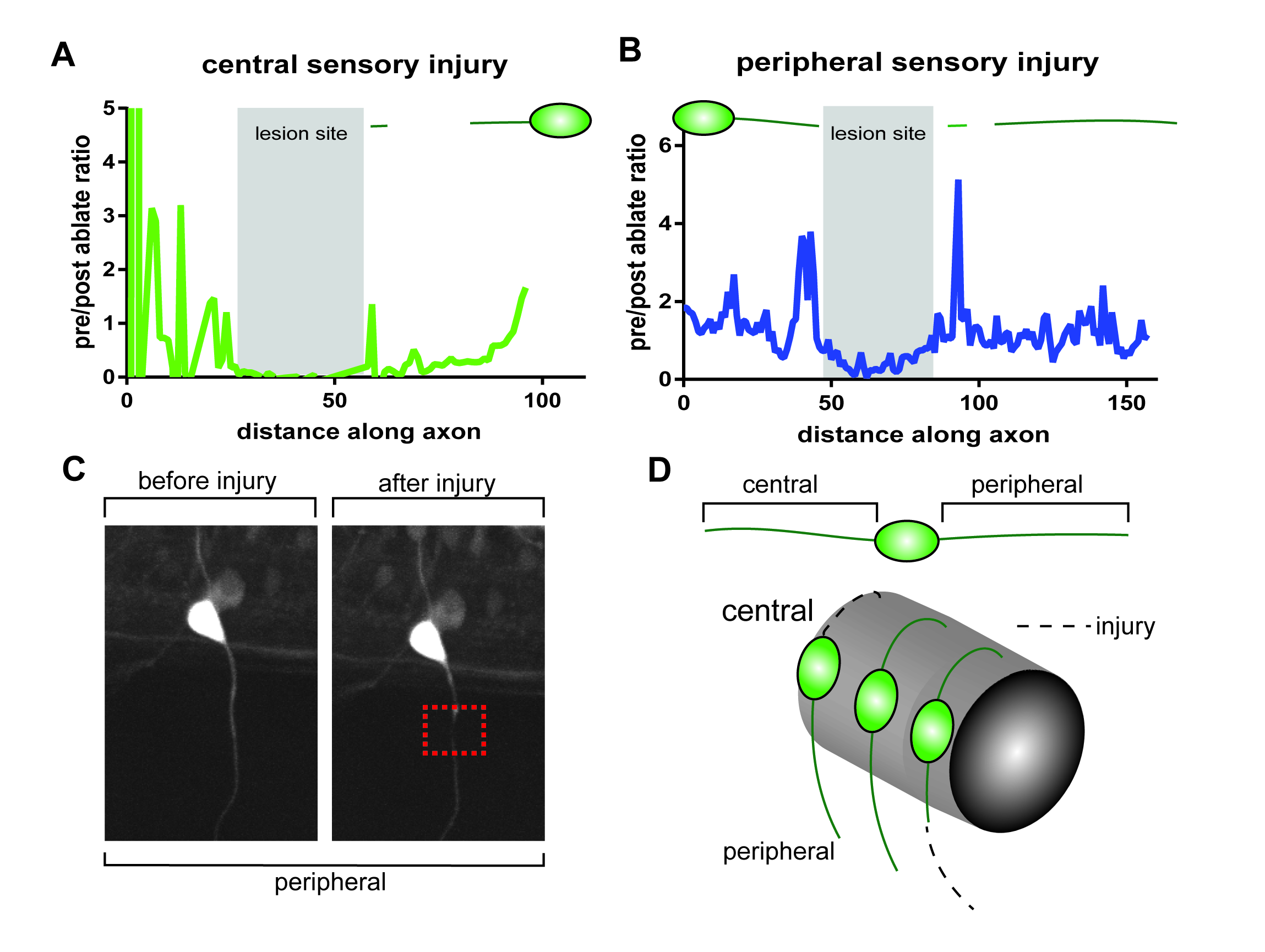

Supplement: S1 Fig — (A) Quantification of decrease in fluorescent signal along the central sensory DRG projection post-injury in Tg(ngn1:gfp) zebrafish at 4 dpf. Gray box indicates lesion site. (B) Schematic of laser-induced avulsion model involving central and peripheral injury. (C) Quantification of decrease in fluorescent signal along the peripheral sensory DRG projection post-injury in Tg(ngn1:gfp) zebrafish at 4 dpf. Gray box indicates lesion site. (D) Confocal z-projection of Tg(ngn1:gfp) zebrafish at 4 dpf showing peripheral injury post-injury. Note that the lesion is specific to the laser exposure site. Red boxes indicate injury site. Scale bar equals 10 μm (D). See S5 Data for raw data. CNS, central nervous system; dpf, days post fertilization; DRG, dorsal root ganglia; PNS, peripheral nervous system. (TIF) [file pbio.3000159.s003.tif]

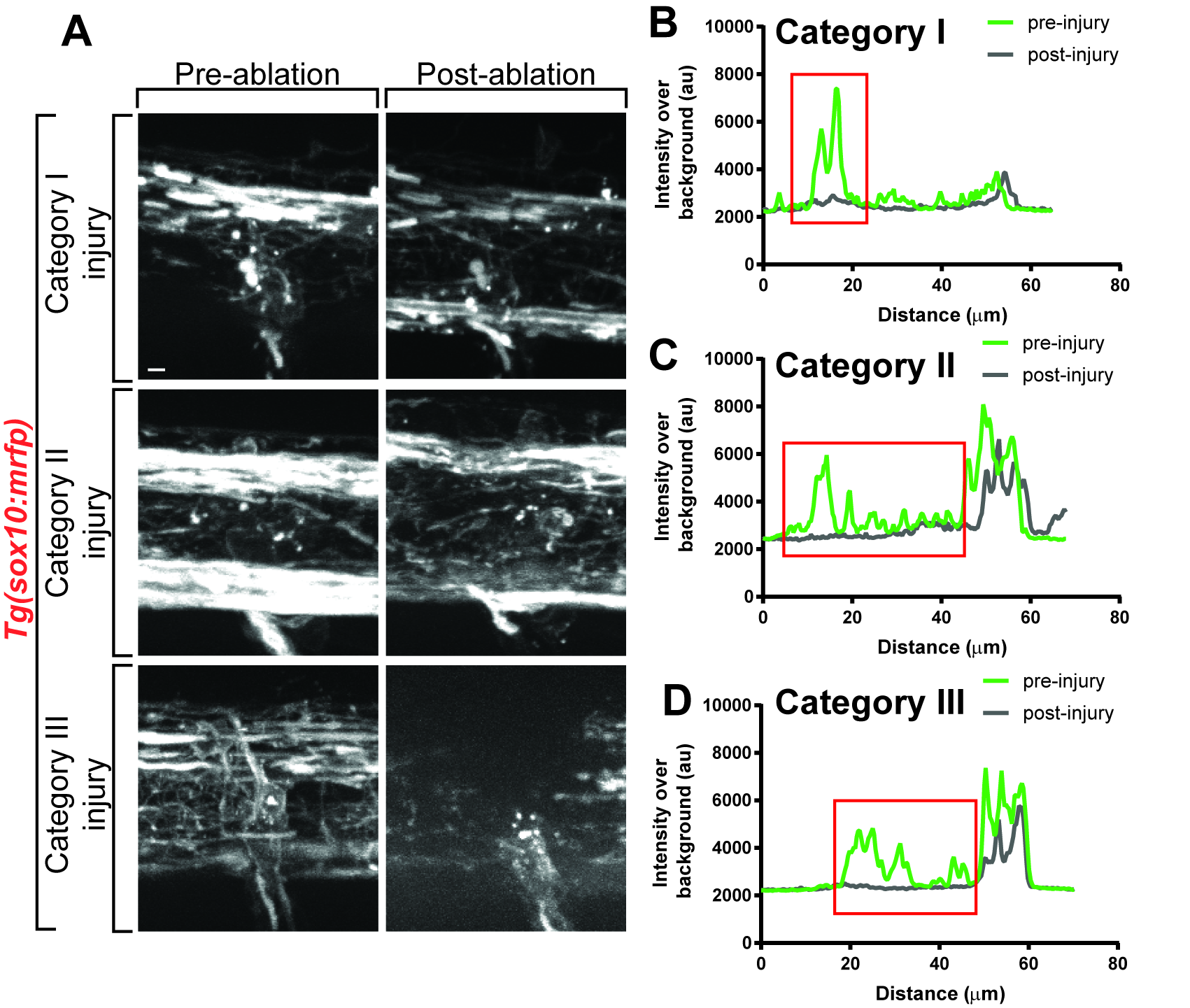

Supplement: S2 Fig — (A) Confocal z-projections of Tg(sox10:mrfp) zebrafish 4 dpf pre- and post-ablation to create category I, II, or III injuries. Qualifications for injury categorization listed in S2 Table. (B) Representative quantification of the intensity over background pre- and post-category I injury. (C) Representative quantification of the intensity over background pre- and post-category II injury. (D) Representative quantification of the intensity over background pre- and post-category III injury. Also, see S2 Table for specific categorical injury parameters. Scale bar equals 10 μm (A). See S6 Data for raw data. dpf, days post fertilization. (TIF) [file pbio.3000159.s004.tif]

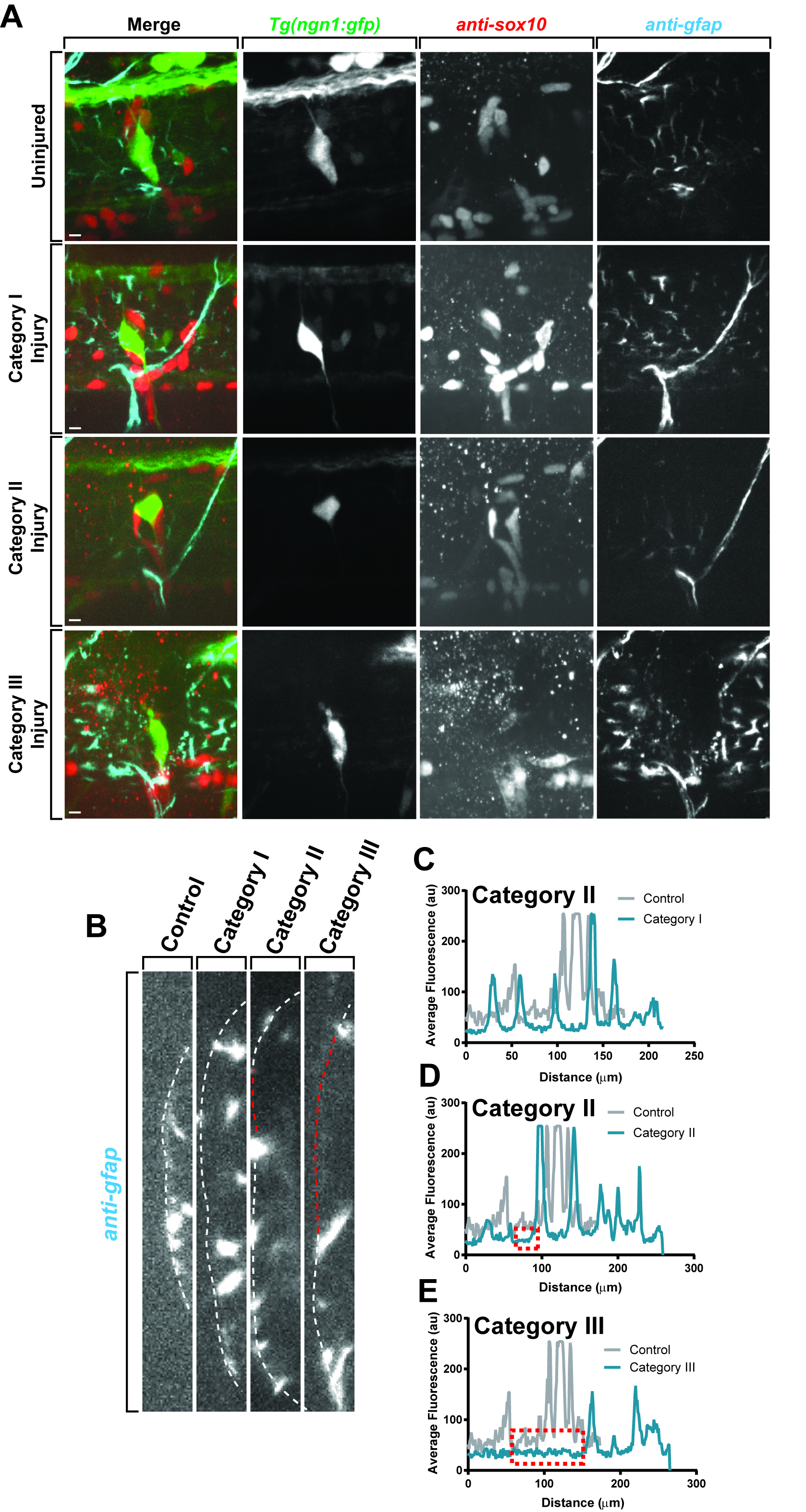

Supplement: S3 Fig — (A) Confocal z-stack images taken at 4 dpf in Tg(ngn1:gfp) zebrafish stained with anti-GFAP and anti-Sox10 antibodies comparing the integrity of spinal cord boundary across all injury categories. (B) Orthogonal rotation view of Tg(pu1:gfp);Tg(sox10:mrfp) animals stained with anti-GFAP showing the GFAP+ boundary of the spinal cord after each injury category. Red dashed line indicates absence of GFAP. (C–E) Quantification of the average fluorescence of GFAP present in control vs category I (C), II (D), and III (E) injuries. Red box equals gfap absence. Scale bar equals 10 μm (A). See S7 Data for raw data. dpf, days post fertilization; GFAP, glial fibrillary acidic protein. (TIF) [file pbio.3000159.s005.tif]

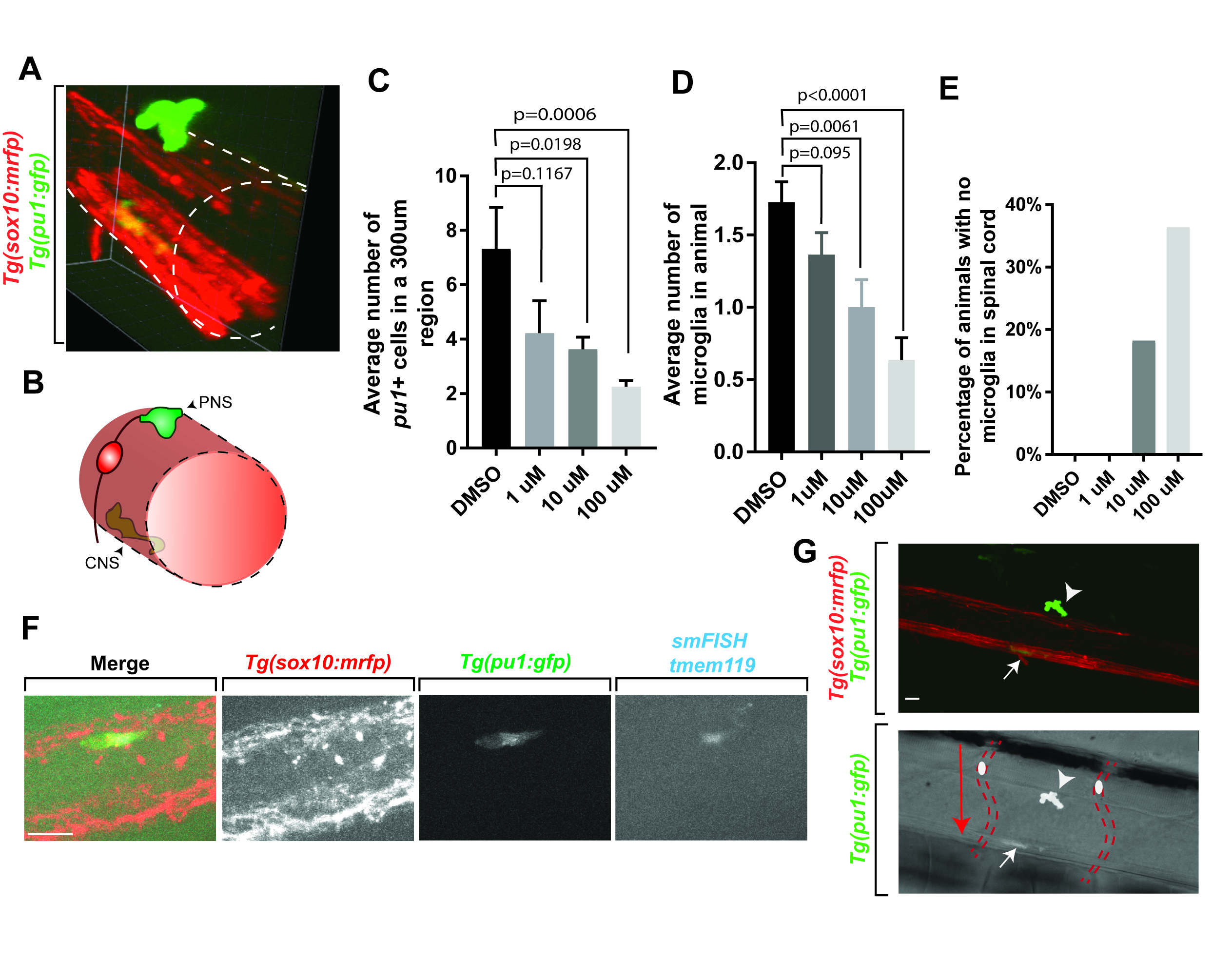

Supplement: S4 Fig — (A) Rotated orthogonal view image from a 24-hour time-lapse movie using Tg(pu1:gfp);Tg(sox10:mrfp) zebrafish at 4 dpf showing microglia inside the spinal cord and a macrophage outside the spinal cord. Dotted lines indicate spinal cord boundary. (B) Graphical representation of 3D image described in (A). (C) Quantification of average number of pu1+ cells present per 300 μm region post-treatment with various GW2580 drug concentrations. (D) Quantification of average number of microglia present in the animal upon GW2580 treatments. (E) Quantification of the percentage of animals with no microglia in the spinal cord upon treatment with GW2580. (F) Confocal z-stack images taken from a Tg(pu1:gfp);Tg(sox10:mrfp) animal stained with smFISH tmem119. (G) Images from a 30-minute time-lapse movie starting at 4 dpf in Tg(pu1:gfp);Tg(sox10:mrfp) zebrafish showing that microglia are not associated with vasculature. Arrows indicate microglia. Arrowheads indicate macrophages in vasculature. Dashed lines indicate blood vessels. Scale bar equals 10 μm (F, G). See S8 Data for raw data. dpf, days post fertilization. (TIF) [file pbio.3000159.s006.tif]

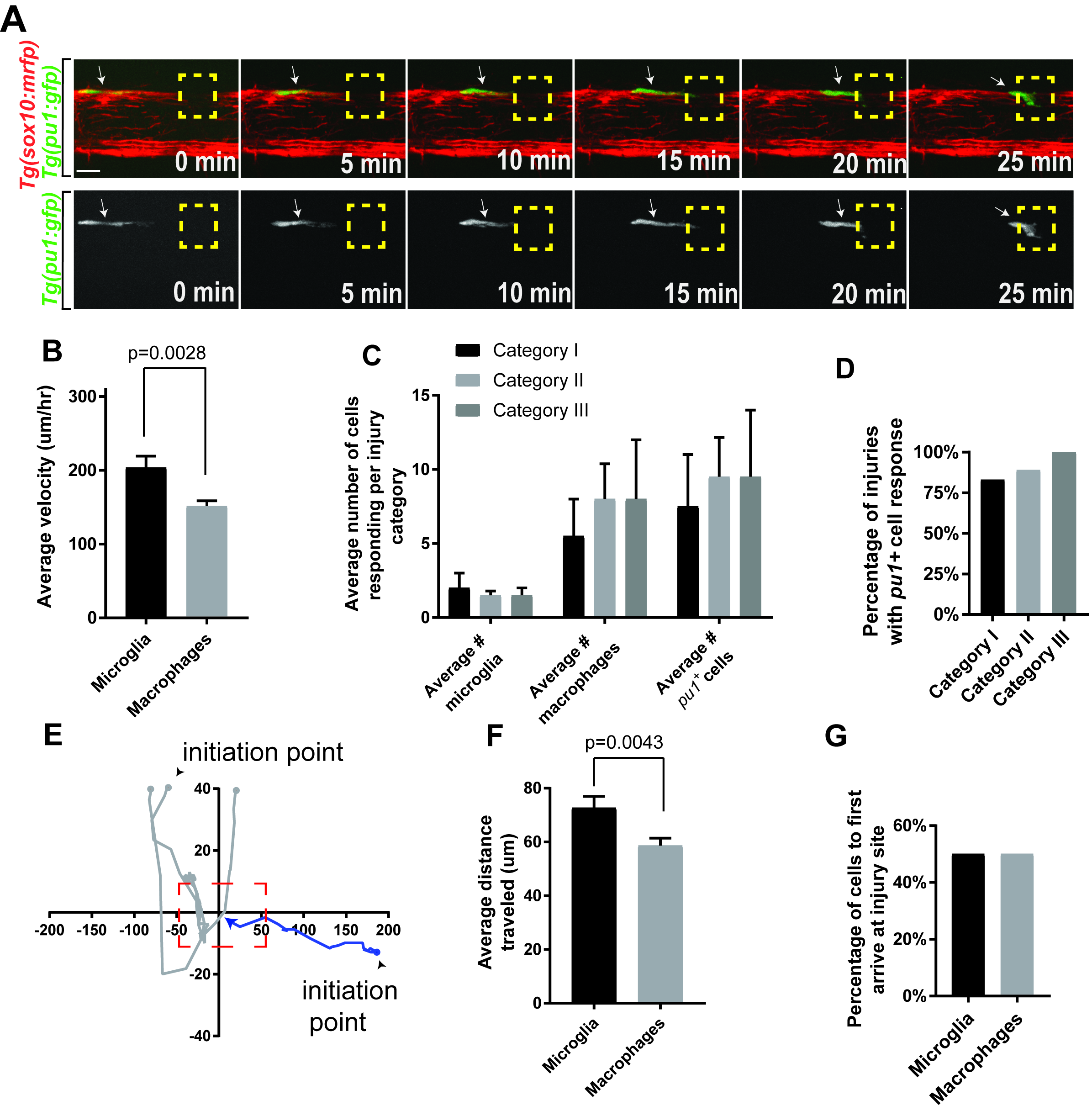

Supplement: S5 Fig — (A) Images from a 24-hour time-lapse movie starting at 4 dpf in Tg(pu1:gfp);Tg(sox10:mrfp) zebrafish showing microglia responding to injury. (B) Quantification of the average velocity of injury response between microglia and macrophages. (C) Quantification of the average number of microglia or macrophages responding to each injury category. (D) Quantification of the percentage of macrophages and microglia the respond to each injury category. (E) Representative migration plot of three macrophages (grey) and one microglia (blue) displaying response of both cells to injury site. (F) Quantification of individual distances microglia and macrophages traveled from their original location to the injury site. (G) Quantification of percentage of phagocytic cells first to arrive at injury site. Scale bar equals 10 μm (A). See S9 Data for raw data. dpf, days post fertilization. (TIF) [file pbio.3000159.s007.tif]

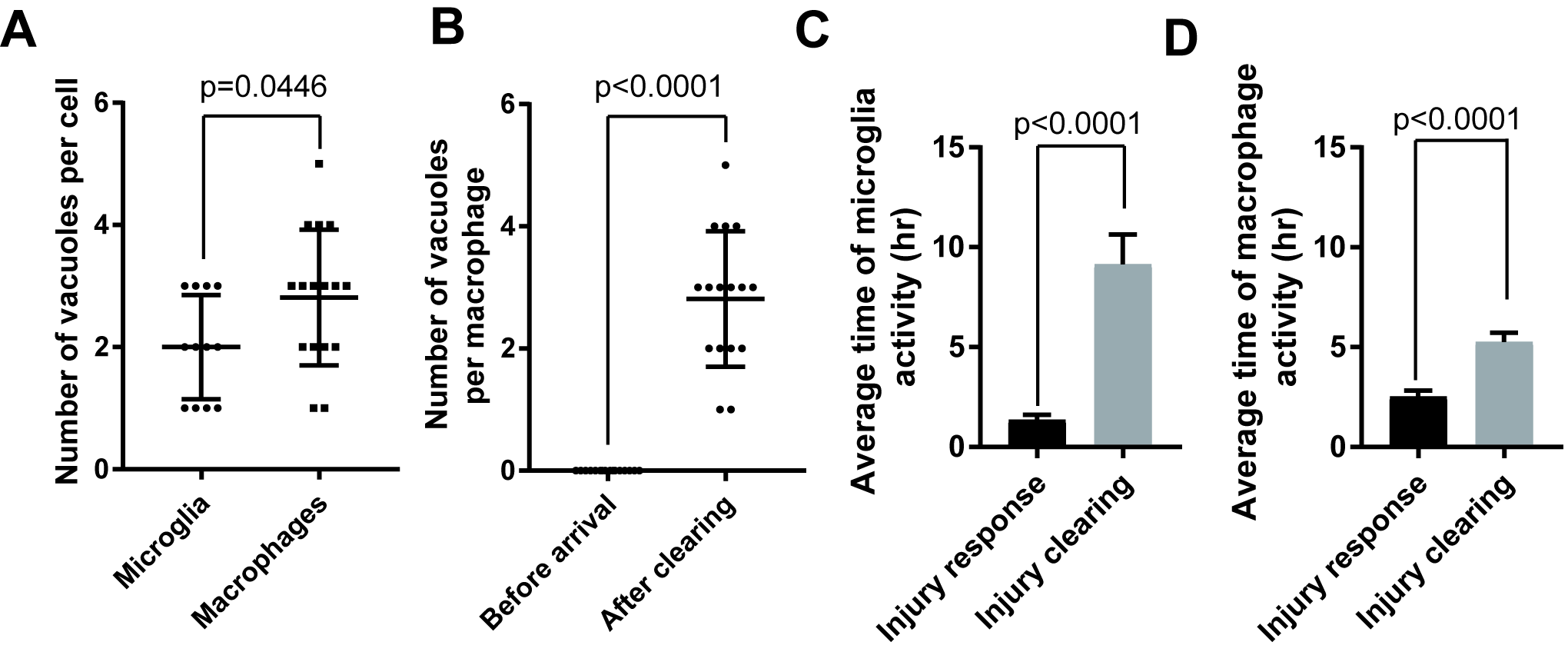

Supplement: S6 Fig — (A) Quantification of individual vacuoles per microglia and macrophage. (B) Quantification of individual vacuoles per macrophage before and during injury response. (C) Quantification of average time microglia spend responding to and clearing injury. (D) Quantification of amount of time macrophages spend responding to and clearing injury. See S10 Data for raw data. (TIF) [file pbio.3000159.s008.tif]

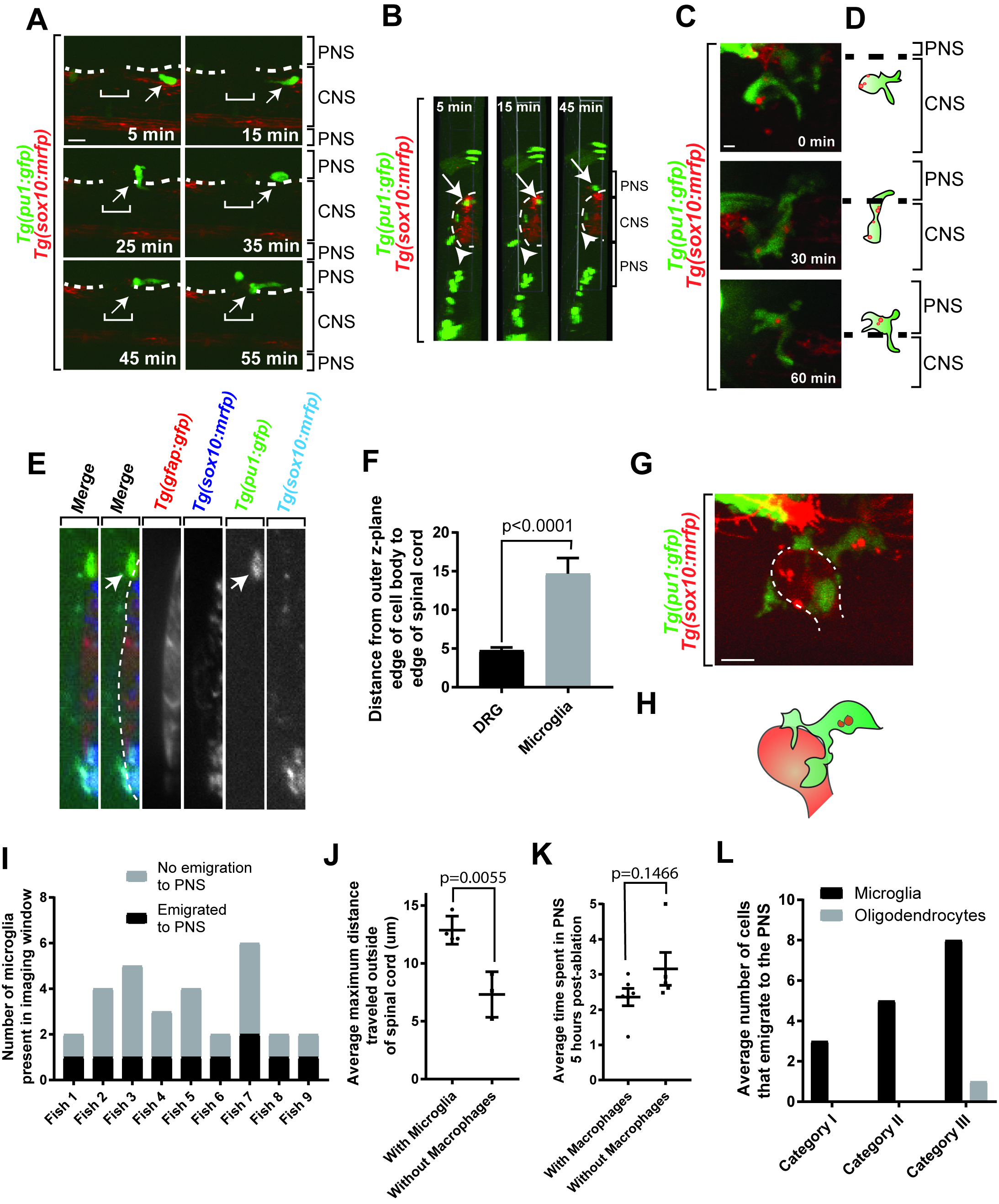

Supplement: S7 Fig — (A) Images from a 24-hour time-lapse movie starting at 4 dpf in Tg(pu1:gfp);Tg(sox10:mrfp) zebrafish showing microglia exiting the CNS. (B) Orthogonal rotation view of Tg(pu1:gfp);Tg(sox10:mrfp) animals at 4 dpf with microglia present outside of the CNS. Arrows indicate microglia. Arrowheads indicate macrophages. Dashed line indicates spinal cord boundary. (C) Images from a 24-hour time-lapse movie starting at 4 dpf in Tg(pu1:gfp);Tg(sox10:mrfp) zebrafish showing microglia squeeze through the injury site. (D) Tracings of ectopically migrating microglia cells described in (C). (E) Overlayed confocal z-stack images from a Tg(gfap:gfp);Tg(sox10:mrfp) animal and a Tg(pu1:gfp);Tg(sox10:mrfp) animal showing the presence of microglia outside of the glial limitans. (F) Quantification of the distance from the outer z-plane edge of the DRG or microglia cell body to the edge of the spinal cord. (G) Confocal image and (H) traced schematic of an excerpt from a time-lapse movie following injury showing microglia in contact with PNS-located DRG cell bodies. (I) Quantification of the number of microglia per animal that are present in the imaging window that did or did not emigrate to the PNS. (J) Quantification of maximum distance ectopic microglia traveled outside of the sox10+ CNS. (K) Quantification of time microglia spent in PNS five hours post-ablation. (L) Quantification of the average number of oligodendrocytes and microglia that are present in the PNS at the site of avulsion. Scale bar equals 1μm (C) and 10 μm (A,G). See S11 Data for raw data. CNS, central nervous system; dpf, days post fertilization; DRG, dorsal root ganglia; PNS, peripheral nervous system. (TIF) [file pbio.3000159.s009.tif]

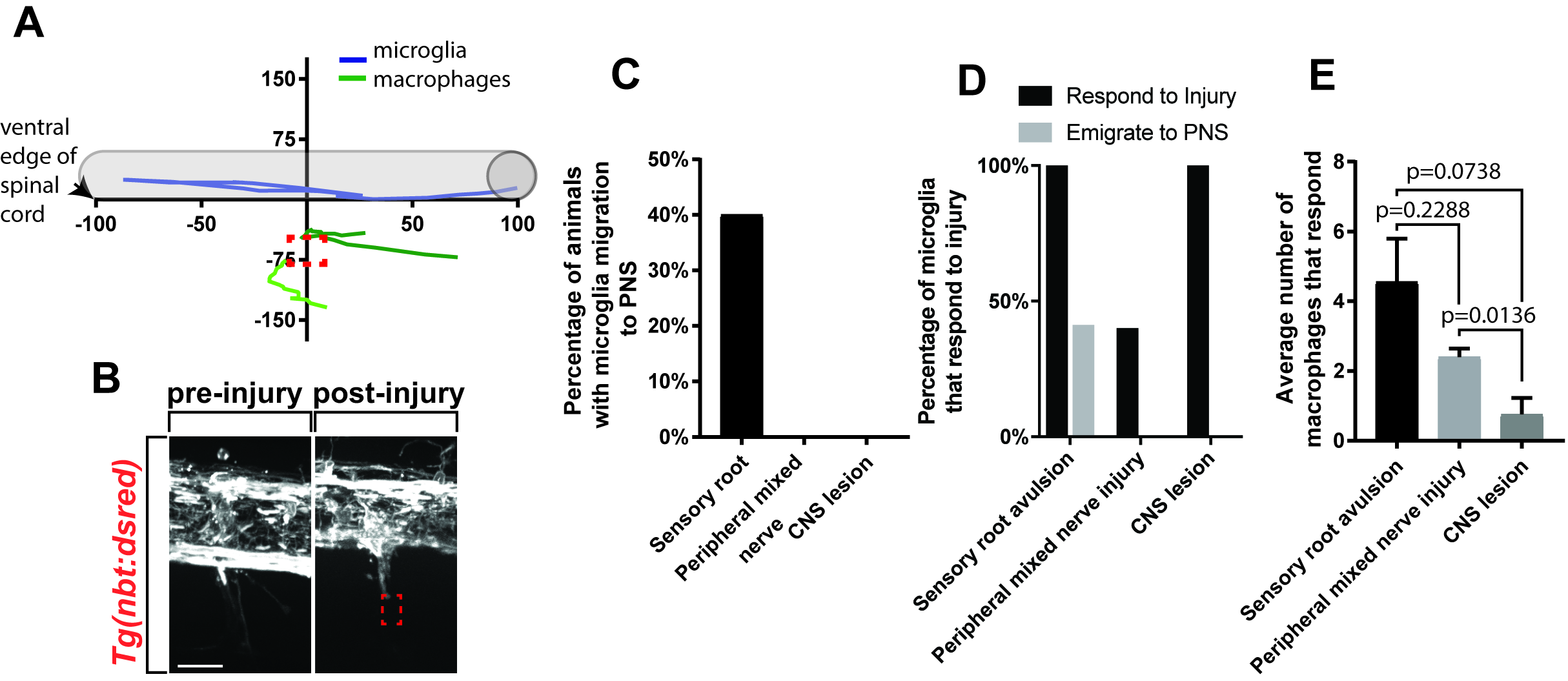

Supplement: S8 Fig — (A) Representative migration plot of two macrophages (green) and one microglia (blue) displaying response of macrophages only to the site of distal peripheral injury. Red box indicates injury site. (B) Confocal z-projection of Tg(nbt:dsred) zebrafish 4 dpf pre- and post-distal peripheral ablation. (C) Quantification of the percentage of movies comparing microglia migration to sensory root avulsion, peripheral mixed nerve avulsion, and CNS-specific injury. (D) Quantification of the average number of microglia present in the imaging window compared with those that respond to peripheral mixed nerve injury or CNS-specific injury. (E) Quantification of the average number of macrophages present in the imaging window compared to those that respond to peripheral mixed nerve injury or CNS-specific injury. Scale bar equals 10 μm (B). See S12 Data for raw data. CNS, central nervous system; dpf, days post fertilization. (TIF) [file pbio.3000159.s010.tif]

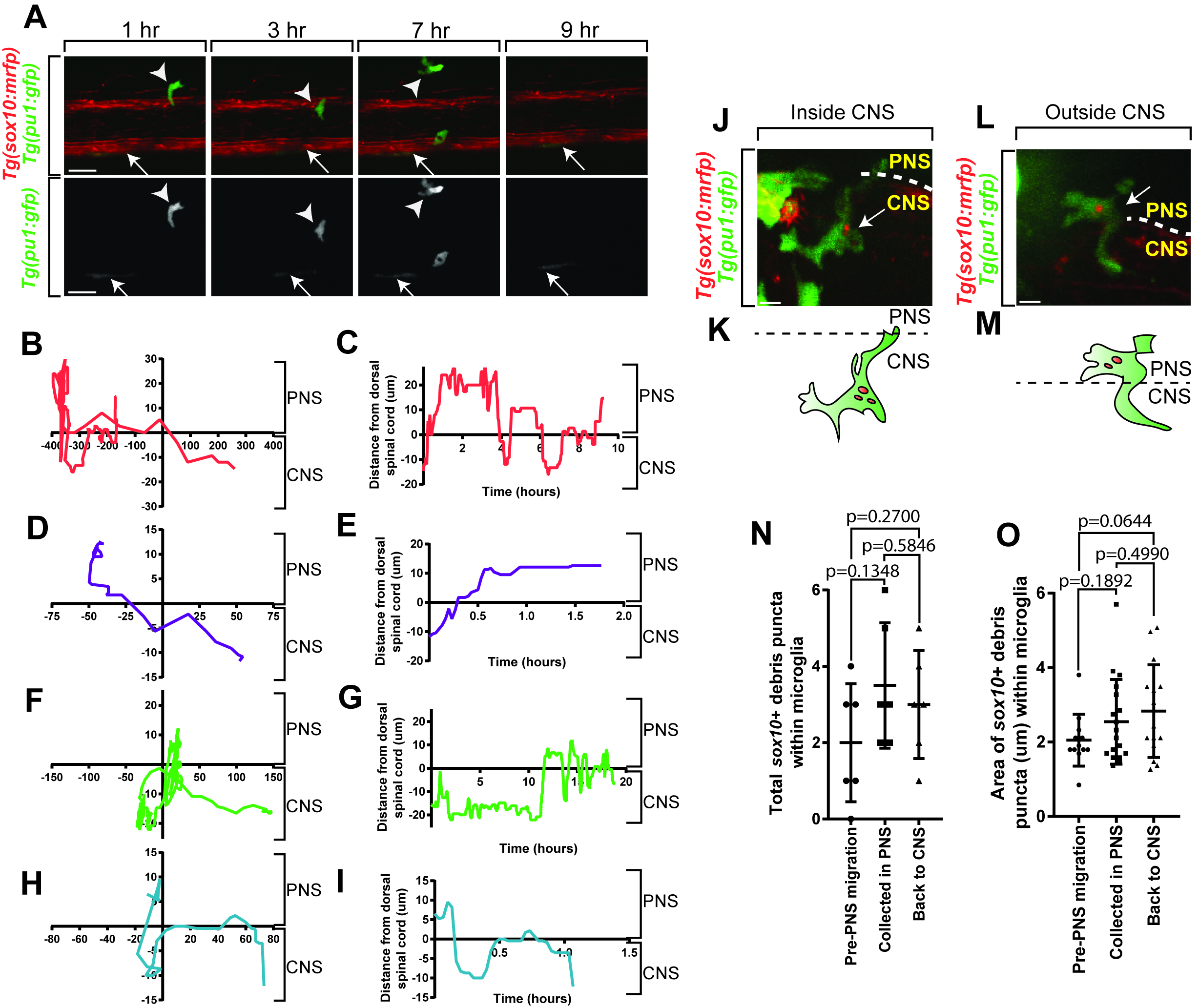

Supplement: S9 Fig — (A) Images from a 24-hour time-lapse movie starting at 4 dpf in Tg(pu1:gfp);Tg(sox10:mrfp) zebrafish showing microglia and macrophages in their typical domains pre-injury. Arrows indicate microglia. Arrowheads indicate macrophages. (B, D, F, H) Migration plots of individual microglia that have migrated to injury site and entered the PNS. (C, E, G, I) Quantification of distance and time a microglia cell spent inside and outside of the CNS. y-axis numbers > 0 indicate cell’s presence in PNS. y-axis numbers < 0 indicate cell’s presence in CNS. (J) Images from a 24-hour time-lapse movie starting at 4 dpf in Tg(pu1:gfp);Tg(sox10:mrfp) zebrafish showing pre-ectopic PNS migration of microglia. Arrows indicate microglia. Dotted line indicates dorsal edge of spinal cord. (K) Tracing of pre-ectopic microglia in (J). (L) Images from a 24-hour time-lapse movie starting at 4 dpf in Tg(pu1:gfp);Tg(sox10:mrfp) zebrafish showing migration of microglia to PNS. Arrows indicate microglia. Dotted line indicates dorsal edge of spinal cord. (M) Tracing of pre-ectopic microglia in (L). (N) Quantification of debris puncta within microglia collected before migration, during migration, and after migration. (O) Quantification of size of debris puncta within microglia collected before migration, during emigration, and after emigration. Scale bar equals 1 μm (J,L) and 10 μm (A). See S13 Data for raw data. CNS, central nervous system; dpf, days post fertilization; PNS, peripheral nervous system. (TIF) [file pbio.3000159.s011.tif]

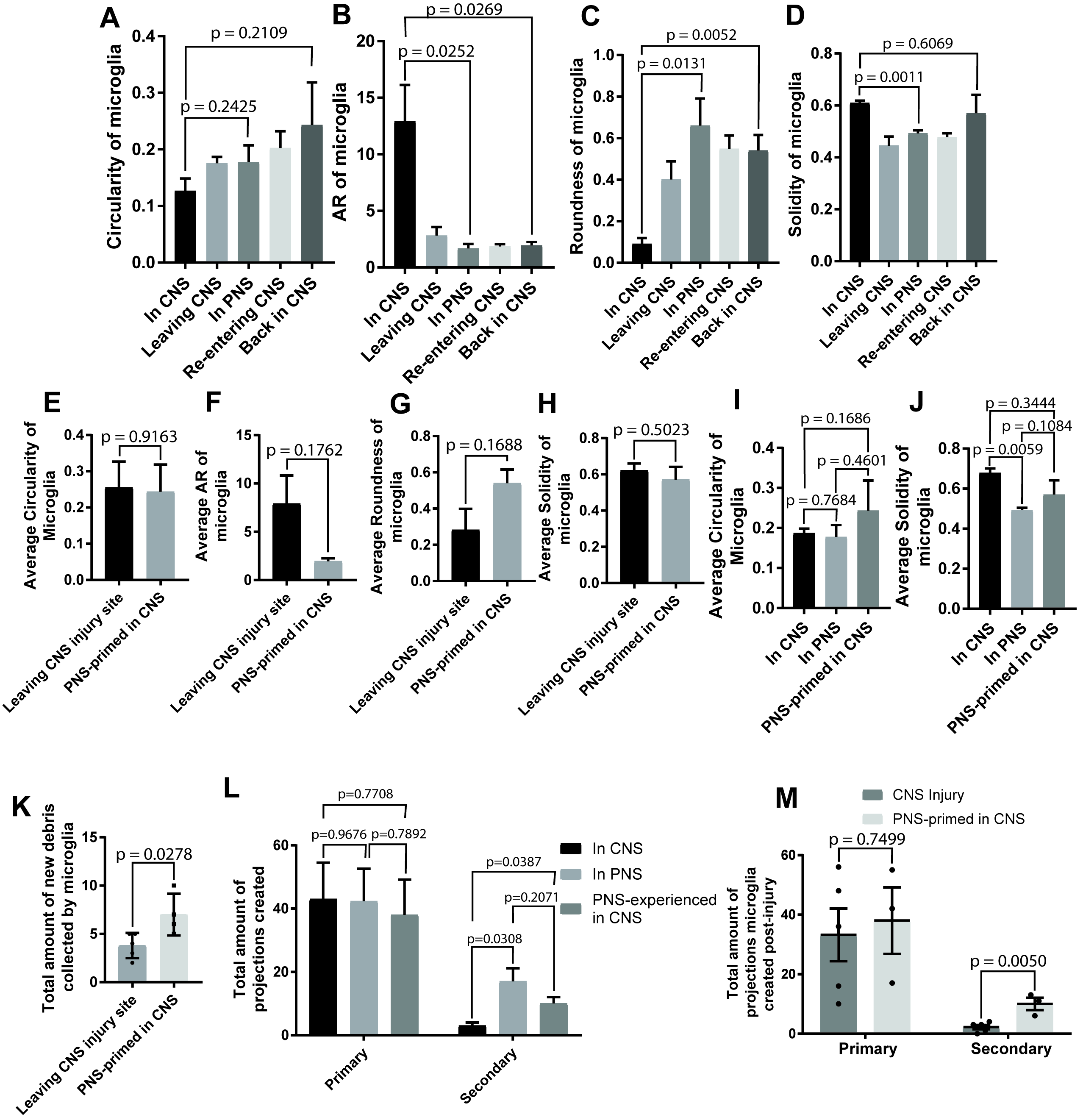

Supplement: S10 Fig — (A–D) Shape descriptor quantification of the circularity (A), aspect ratio (B), roundness (C), and solidity (D) of microglia in the CNS, leaving the CNS, in PNS, re-entering the CNS, and PNS-primed in the CNS. (E–I) Quantification of the average circularity (E), aspect ratio (F), roundness (G), and solidity (H) of a PNS-primed microglia in the CNS versus a microglia that responded to injury but never left the CNS. (I) Shape descriptor quantification of the circularity of individual microglia in the CNS, in PNS, and PNS-primed in the CNS. (J) Shape descriptor quantification of the solidity of individual microglia in the CNS, in PNS, and PNS-primed in the CNS. (K) Quantification of the total amount of new debris a PNS-primed microglia collected compared to microglia that responded to injury but never left the CNS. (L) Quantification of the amount of primary and secondary projections created across different neuronal domains. (M) Quantification of the total amount of primary and secondary projections a PNS-primed microglia in the CNS versus a microglia that responded to injury but never left the CNS. See S14 Data for raw data. CNS, central nervous system; PNS, peripheral nervous system. (TIF) [file pbio.3000159.s012.tif]

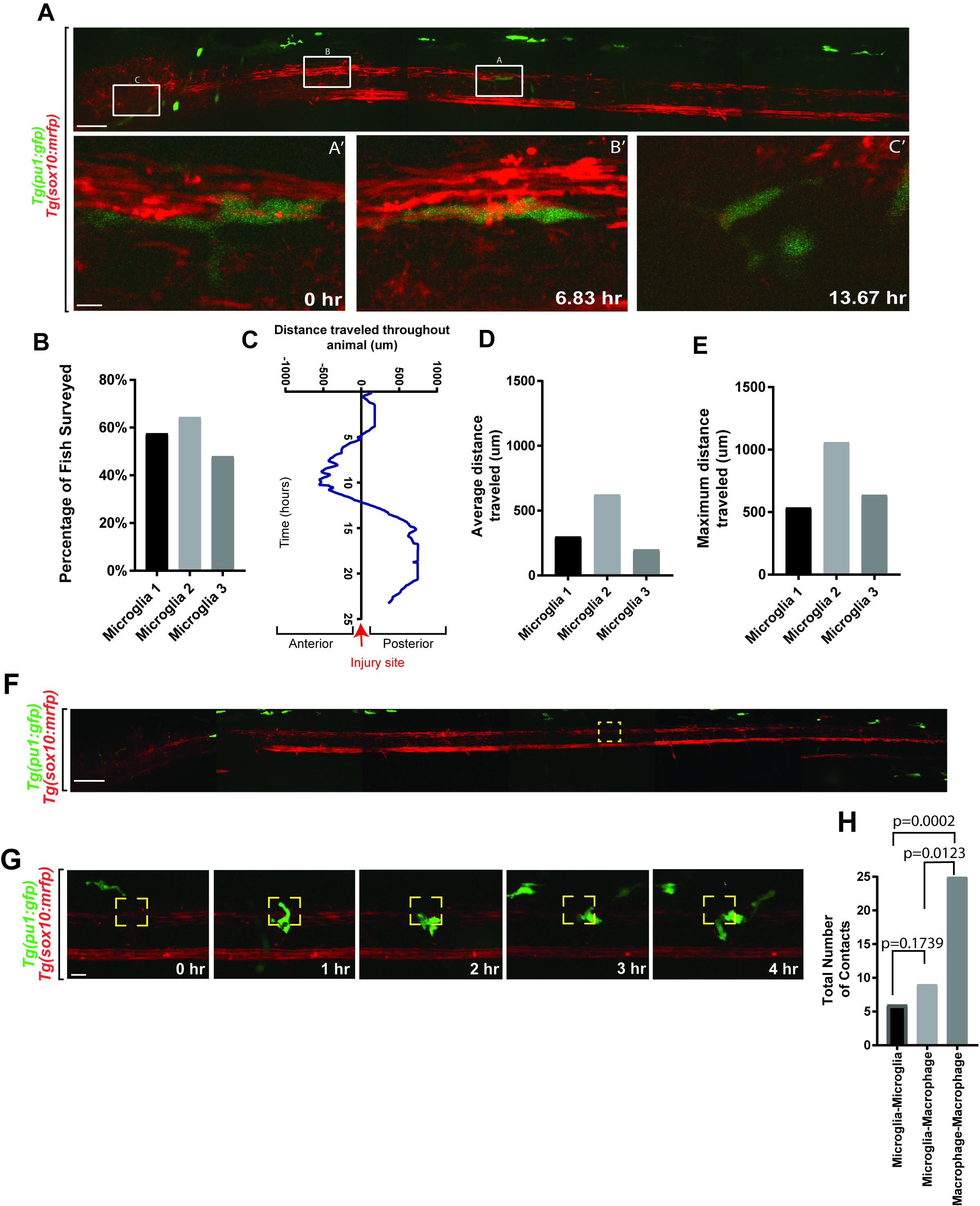

Supplement: S11 Fig — (A) Stitched zoning images from a 24-hour time-lapse movie starting at 4 dpf in Tg(pu1:gfp);Tg(sox10:mrfp) zebrafish representing the entire spinal cord. White boxes coordinate with letter tags represent zones of the animal in which microglia traveled, larger images located below. Yellow box indicates injury site. Note that microglia traveled into brain region shown in (C). (B) Quantification of the percent of the animal surveyed by microglia. (C) Representative quantification of distance microglia traveled throughout the animal anteriorly and posteriorly. Red arrow indicates injury site, origin set to site of injury. (D) Quantification of the average distance each microglia travel throughout the CNS. (E) Quantification of the maximum distance each microglia travel throughout the CNS. (F) Stitched zoning images from a 24-hour time-lapse movie starting at 4 dpf in Tg(pu1:gfp);Tg(sox10:mrfp) zebrafish representing the entire animal and no microglia located within the CNS. Yellow boxes indicate injury site. (G) Images from a 24-hour time-lapse movie starting at 4 dpf in Tg(pu1:gfp);Tg(sox10:mrfp) zebrafish showing macrophage response to injury site. Yellow box indicates injury site. (H) Quantification of the total amount of heterotypic and homotypic cellular contacts. Scale bar equals 1 μm (bottom A), 10 μm (G), 100 μm (top A, F). See S15 Data for raw data. CNS, central nervous system; dpf, days post fertilization. (TIF) [file pbio.3000159.s013.tif]

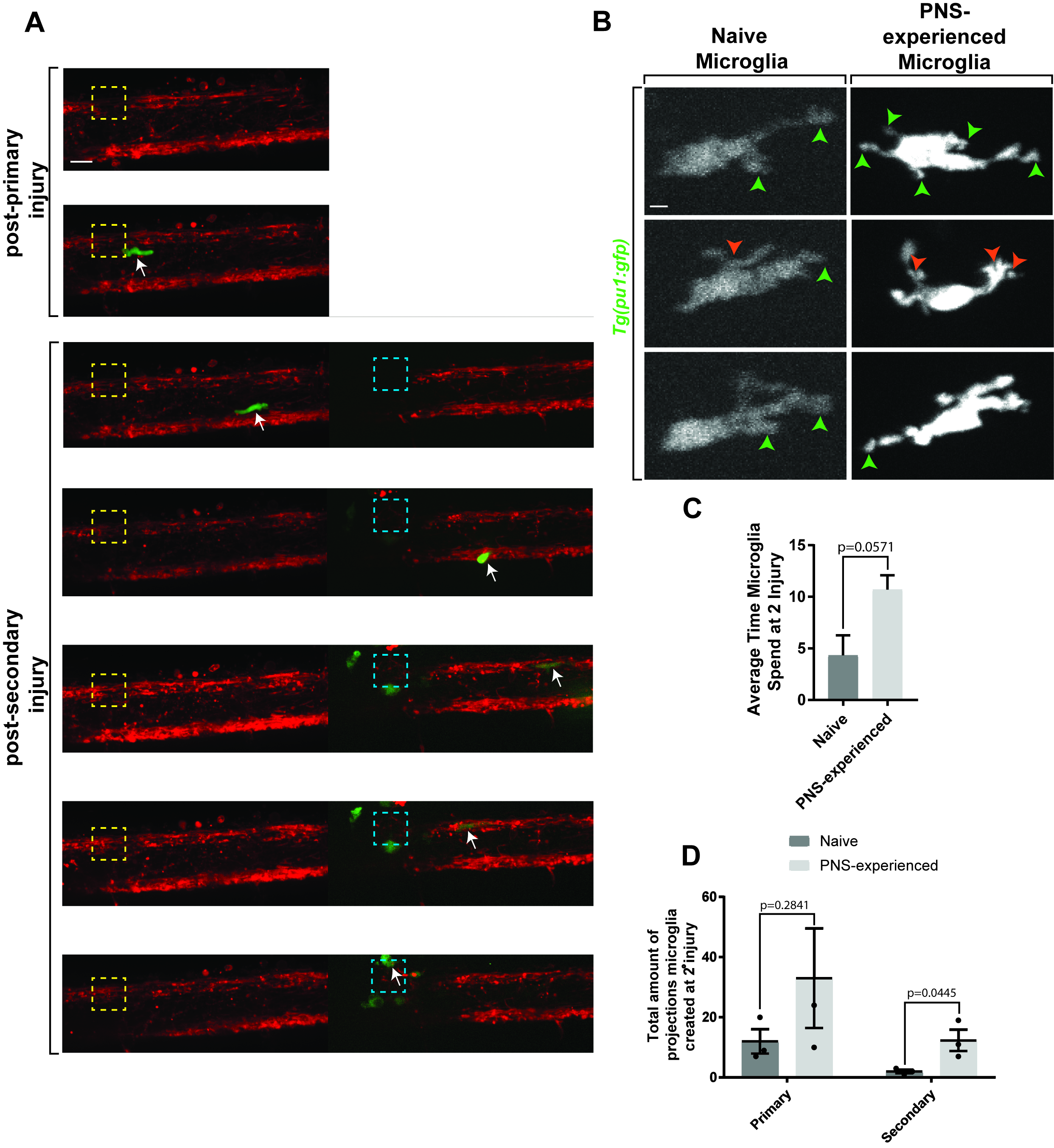

Supplement: S12 Fig — (A) Images from a 24-hour time-lapse movie starting at 4 dpf in Tg(pu1:gfp);Tg(sox10:mrfp) zebrafish showing microglia arriving at the primary injury site, emigrating from the CNS, re-entering the CNS, and then migrating to the secondary injury site. Arrow indicates microglia. Yellow box indicates primary injury site. Blue box indicates secondary injury site. (B) Images from a 24-hour time-lapse movie starting at 4 dpf in Tg(pu1:gfp);Tg(sox10:mrfp) showing primary and secondary projections of PNS-experienced microglia at the secondary injury site. Green arrowheads indicate primary projections. Orange arrowheads indicate secondary projections. (C) Quantification of the average time microglia spent at the secondary injury site. (D) Quantification of the amount of primary and secondary projections PNS-experienced microglia created at the secondary injury site compared to naïve microglia. Scale bar equals 1 μm (B), 10 μm (A). See S16 Data for raw data. CNS, central nervous system; dpf, days post fertilization; PNS, peripheral nervous system. (TIF) [file pbio.3000159.s014.tif]

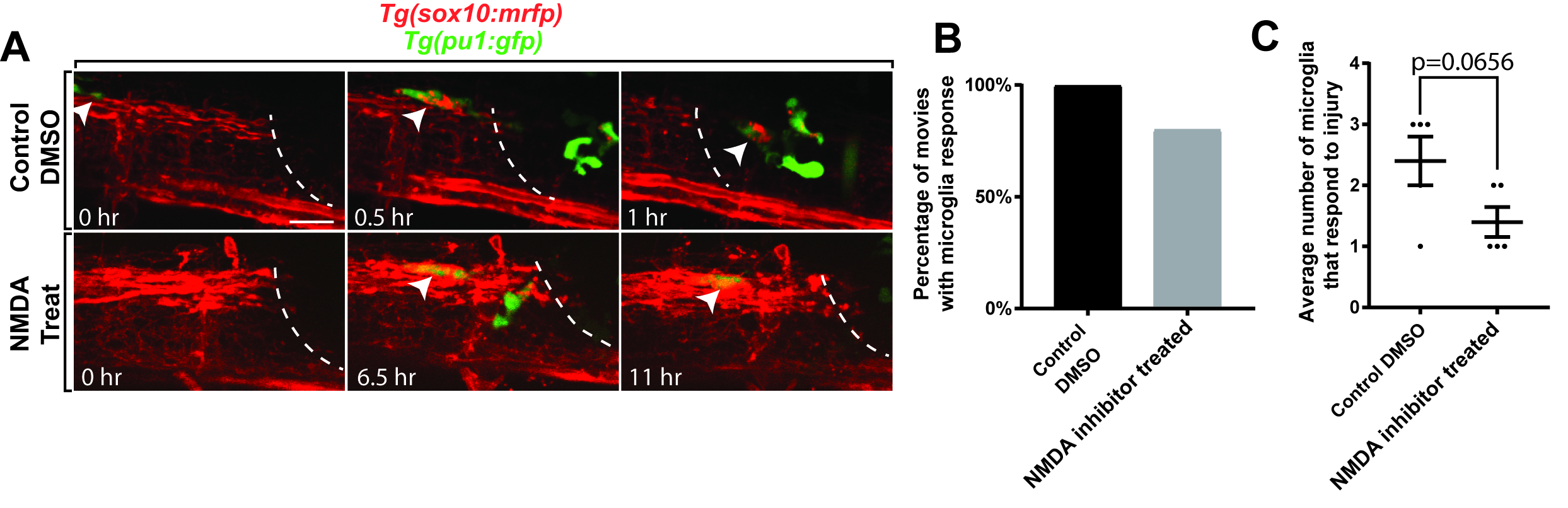

Supplement: S13 Fig — (A) Images from a 24-hour time-lapse movie starting at 4 dpf in Tg(pu1:gfp);Tg(sox10:mrfp) zebrafish comparing DMSO and NMDA inhibitor treated microglia response to the injury site. Arrowheads indicate microglia. (B) Quantification of the percentage of DMSO and NMDA-inhibitor treated animals that had microglia respond to the site of injury. (C) Quantification of the average number of microglia that are present and respond to the injury site. Scale bar equals 10 μm (A). See S17 Data for raw data. dpf, days post fertilization; NMDA, N-methyl-D-aspartate receptor. (TIF) [file pbio.3000159.s015.tif]

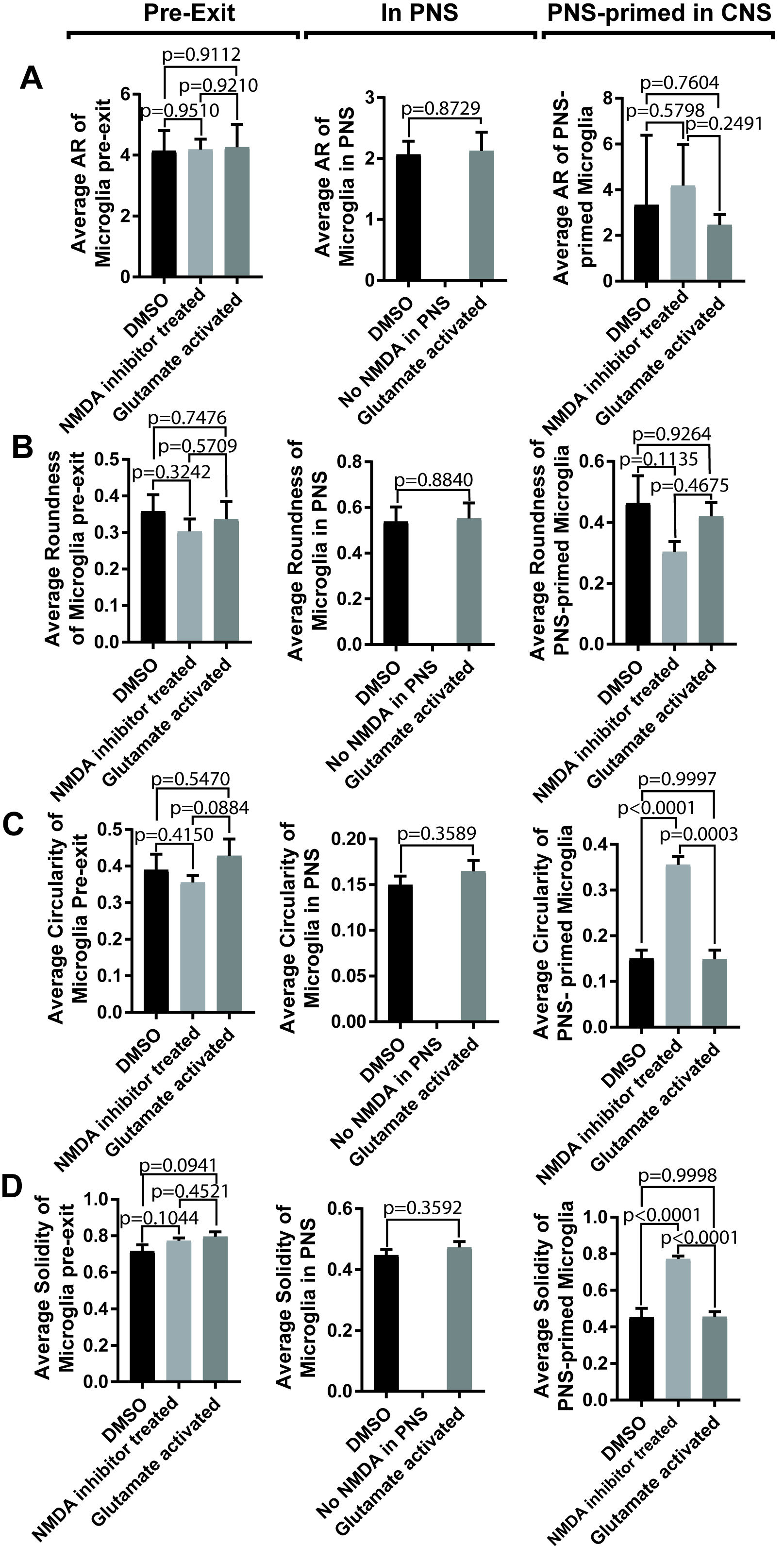

Supplement: S14 Fig — (A-D) Shape descriptor quantification of the circularity (A), aspect ratio (B), roundness (C), and solidity (D) of DMSO compared to NMDA- and glutamate-treated individual microglia before exiting the CNS, in the PNS, and PNS-primed in the CNS. See S18 Data for raw data. CNS, central nervous system; NMDA, N-methyl-D-aspartate receptor; PNS, peripheral nervous system. (TIF) [file pbio.3000159.s016.tif]

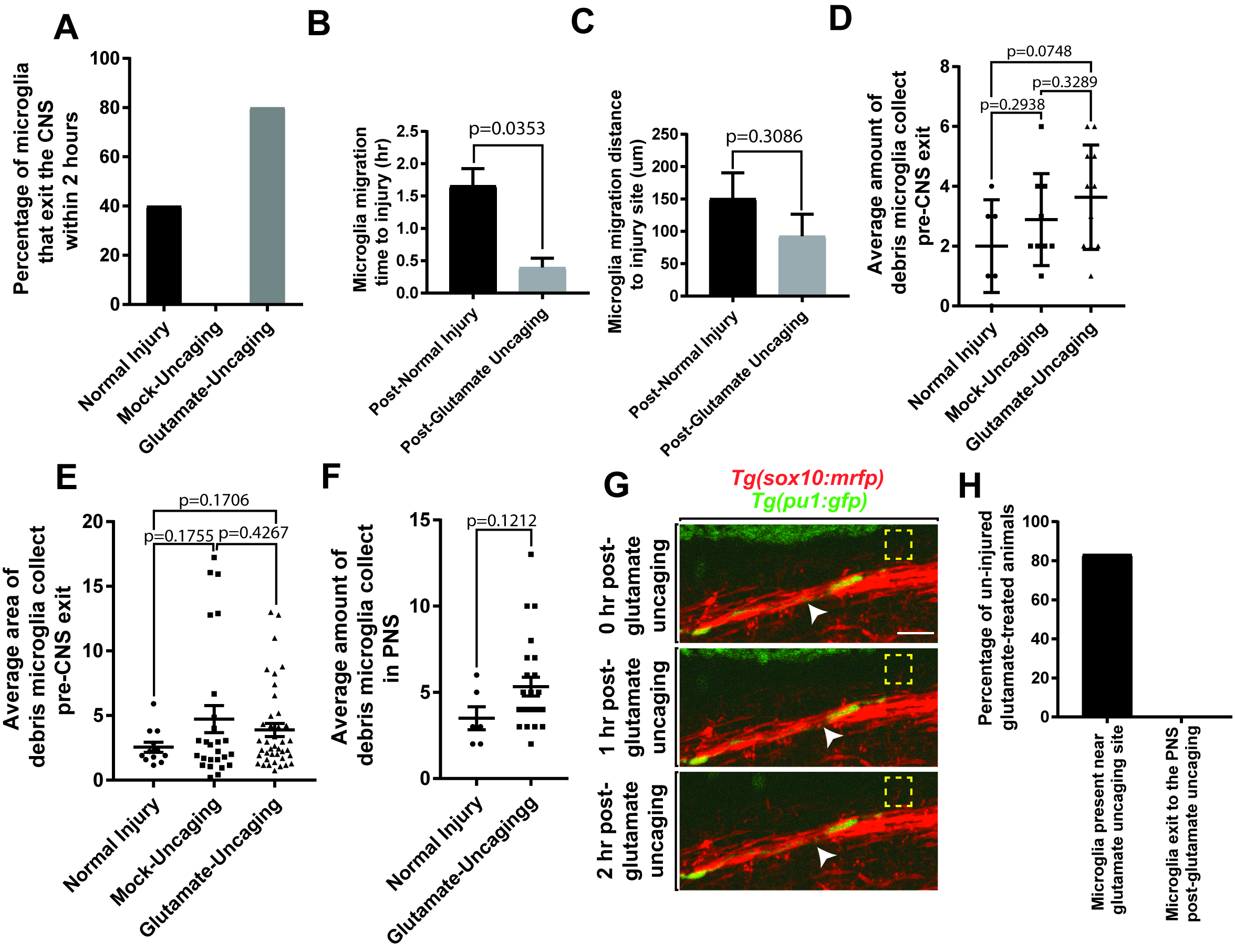

Supplement: S15 Fig — (A) Quantification of the percentage of microglia that exit the CNS within 2 hours post-injury after no glutamate exposure, mock uncaging, and glutamate uncaging. (B) Quantification of the time it takes microglia to respond to injury post-normal injury versus post-glutamate uncaging. (C) Quantification of the distance microglia travel to the injury site post-normal injury versus post-glutamate uncaging. (D) Quantification of the average amount of debris individual microglia collect pre-CNS exit when treated with glutamate. (E) Quantification of the average area of the debris collected by individual microglia pre-CNS exit represented in (D). (F) Quantification of the average amount of debris individual microglia collect while in the PNS when treated with glutamate. (G) Images from a 24-hour time-lapse movie starting at 4 dpf in uninjured glutamate-treated Tg(pu1:gfp);Tg(sox10:mrfp) zebrafish showing no emigration of microglia to the PNS. Arrowheads indicate microglia. Yellow box indicates site of glutamate uncaging. (H) Quantification of the percentage of uninjured glutamate-treated animals with microglia present near the uncaging site compared to the percentage of microglia that exited the CNS following glutamate uncaging. Scale bar equals 10 μm (G). See S19 Data for raw data. CNS, central nervous system; dpf, days post fertilization; PNS, peripheral nervous system. (TIF) [file pbio.3000159.s017.tif]

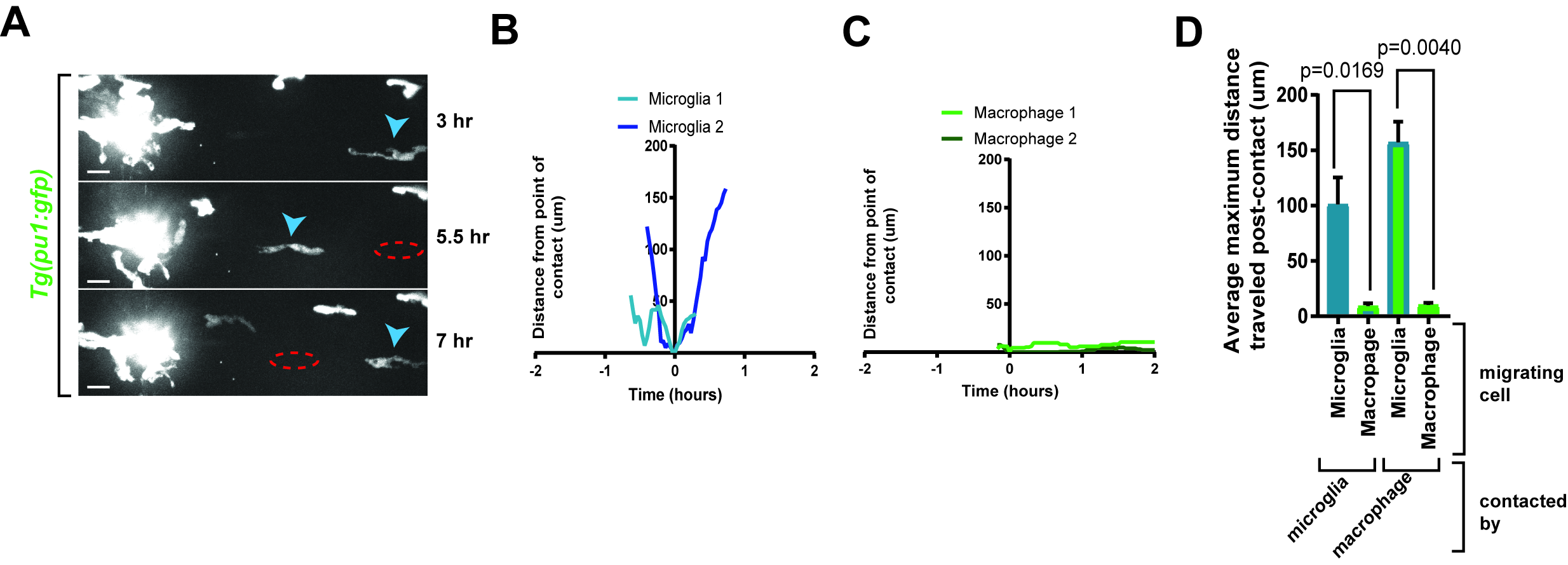

Supplement: S16 Fig — (A) Images from a 24-hour time-lapse movie starting at 4 dpf in Tg(pu1:gfp);Tg(sox10:mrfp) zebrafish showing microglia experience directional changes. Arrowheads indicate microglia. Red circles indicate previous location of microglia. (B) Quantification of distance traveled pre- and post-contact between two microglia. (C) Quantificaion of distance traveled pre- and post-contact between two macrophages. (D) Quantification of average maximum distance traveled of each cell that experiences a directional change pre- and post-contact with a migrating cell (p = 0.0169, p = 0.0040). Scale bar equals 10 μm (A). Stats summarized in S1 Table. See S20 Data for raw data. dpf, days post fertilization. (TIF) [file pbio.3000159.s018.tif]

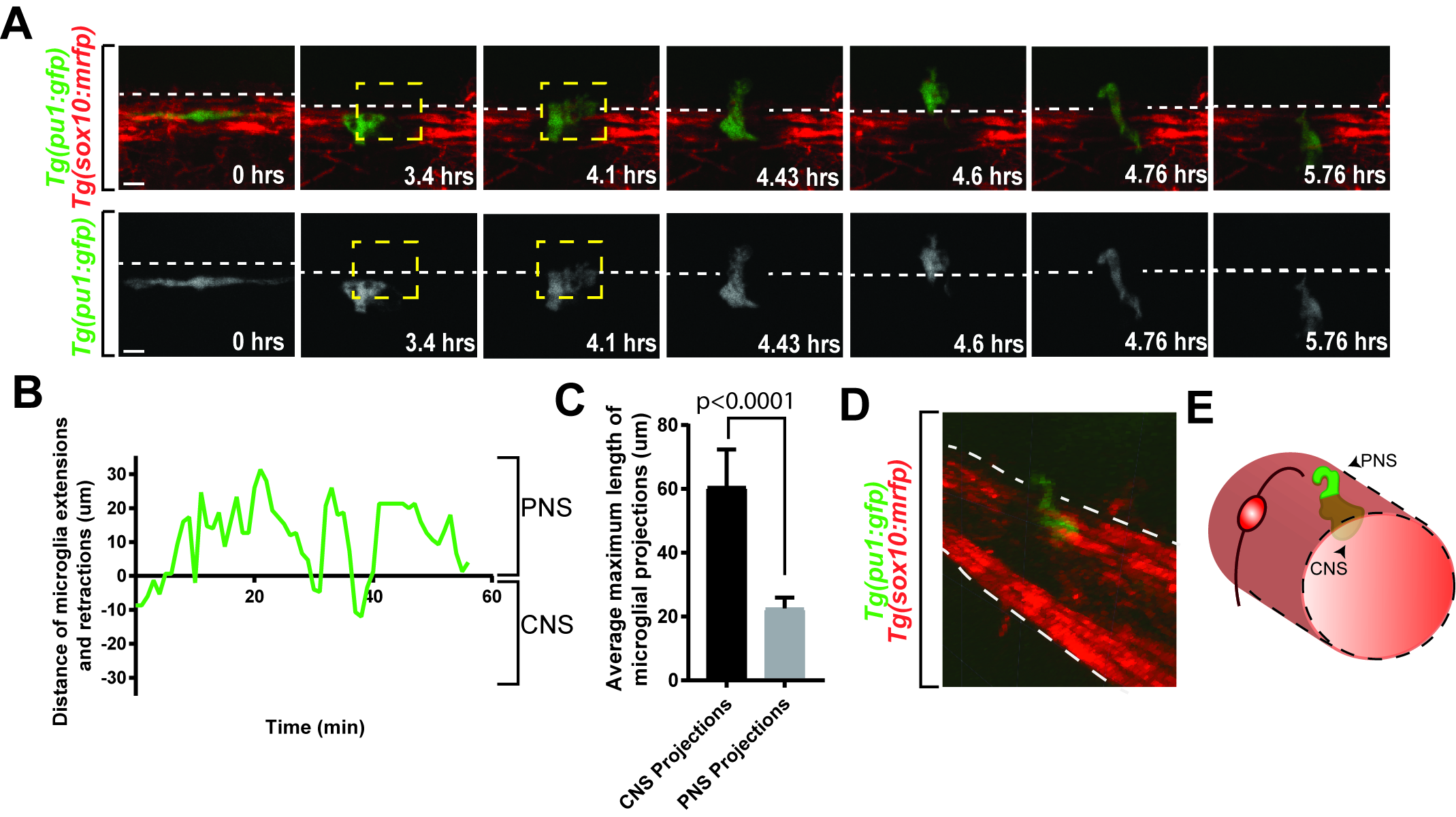

Supplement: S17 Fig — (A) Images from a 6-hour time-lapse movie starting at 4 dpf in Tg(pu1:gfp);Tg(sox10:mrfp) zebrafish showing the ectopic migration of microglia. Dotted line indicates dorsal edge of spinal cord. Yellow box indicates injury site. (B) Quantification of reaching events. y-axis numbers > 0 indicate projection presence in PNS. y-axis numbers < 0 indicate projection presence in CNS. (C) Quantification of microglial projection length in the CNS versus PNS. (D) 3D side view image from a 24-hour time-lapse movie starting at 4 dpf in Tg(pu1:gfp);Tg(sox10:mrfp) zebrafish showing microglial projection reaching into the PNS. (E) Graphical representation of events described in (D). Scale bar equals 1 μm (A). See S21 Data for raw data. CNS, central nervous system; dpf, days post fertilization; PNS, peripheral nervous system. (TIF) [file pbio.3000159.s019.tif]

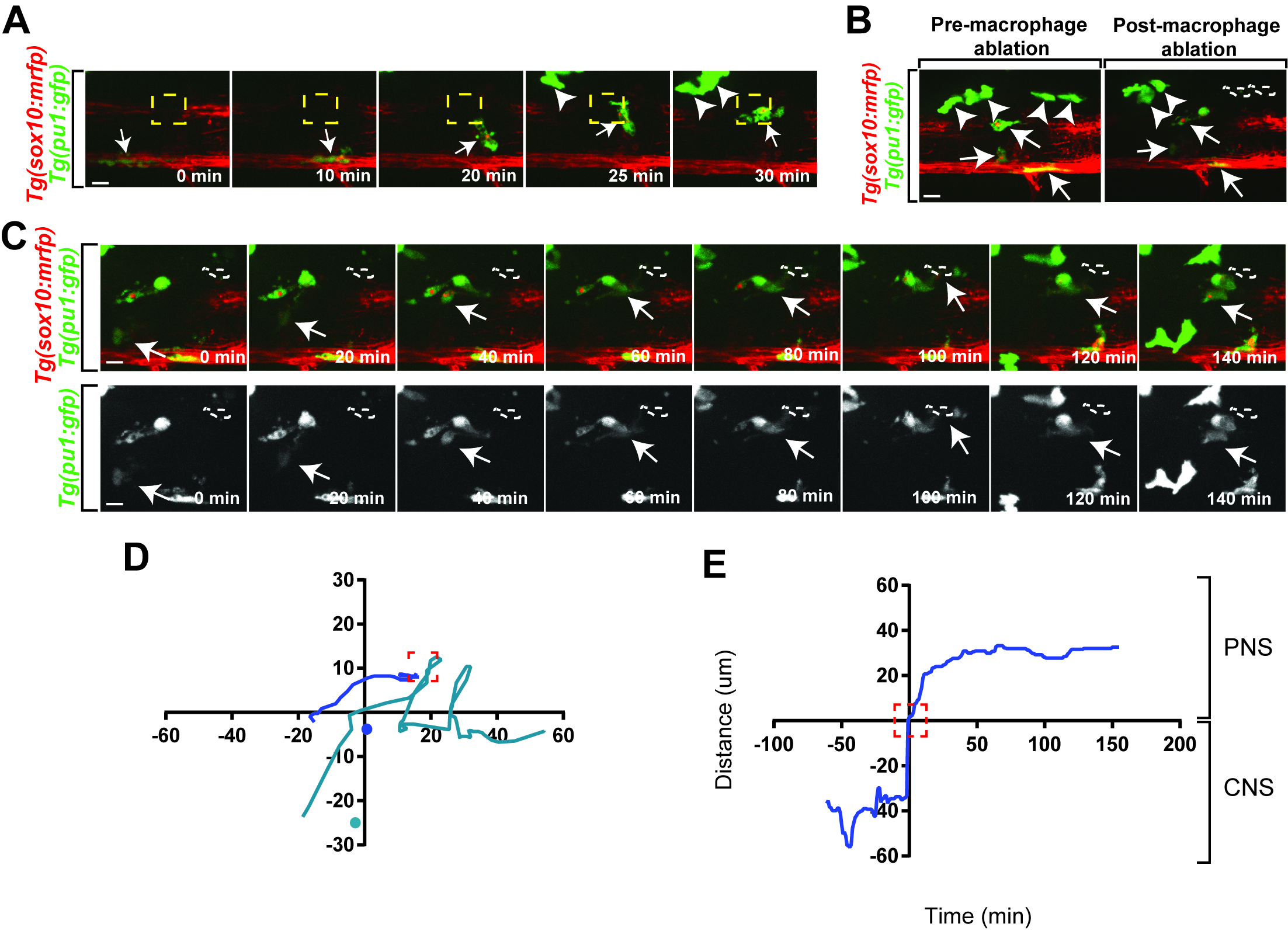

Supplement: S18 Fig — (A) Images from a 2-hour time-lapse movie starting at 4 dpf in Tg(pu1:gfp);Tg(sox10:mrfp) zebrafish post-injury showing pU1+ cell responses to injury. (B) Images from a 2-hour time-lapse movie starting at 4 dpf in Tg(pu1:gfp);Tg(sox10:mrfp) zebrafish before and after single-cell macrophage ablation. Arrows indicate microglia. Arrowheads indicate macrophages. Dashed circles indicate ablated macrophages. (C) Images from 24-hour time-lapse movies starting at 4 dpf in Tg(pu1:gfp);Tg(sox10:mrfp) zebrafish post-macrophage ablation showing microglial response to ablation site. Arrows indicate microglia. Dashed circles indicate site of macrophage ablation. (D) Migration plot representing the migration of microglia directly to the site of macrophage ablation. (E) Quantification of the time and distance microglia traveled immediately following macrophage ablation. Red box indicates injury site. y-axis numbers > 0 indicates the PNS. y-axis numbers < 0 indicates the CNS. Scale bar equals 10 μm (A-C). See S22 Data for raw data. CNS, central nervous system; dpf, days post fertilization; PNS, peripheral nervous system. (TIF) [file pbio.3000159.s020.tif]

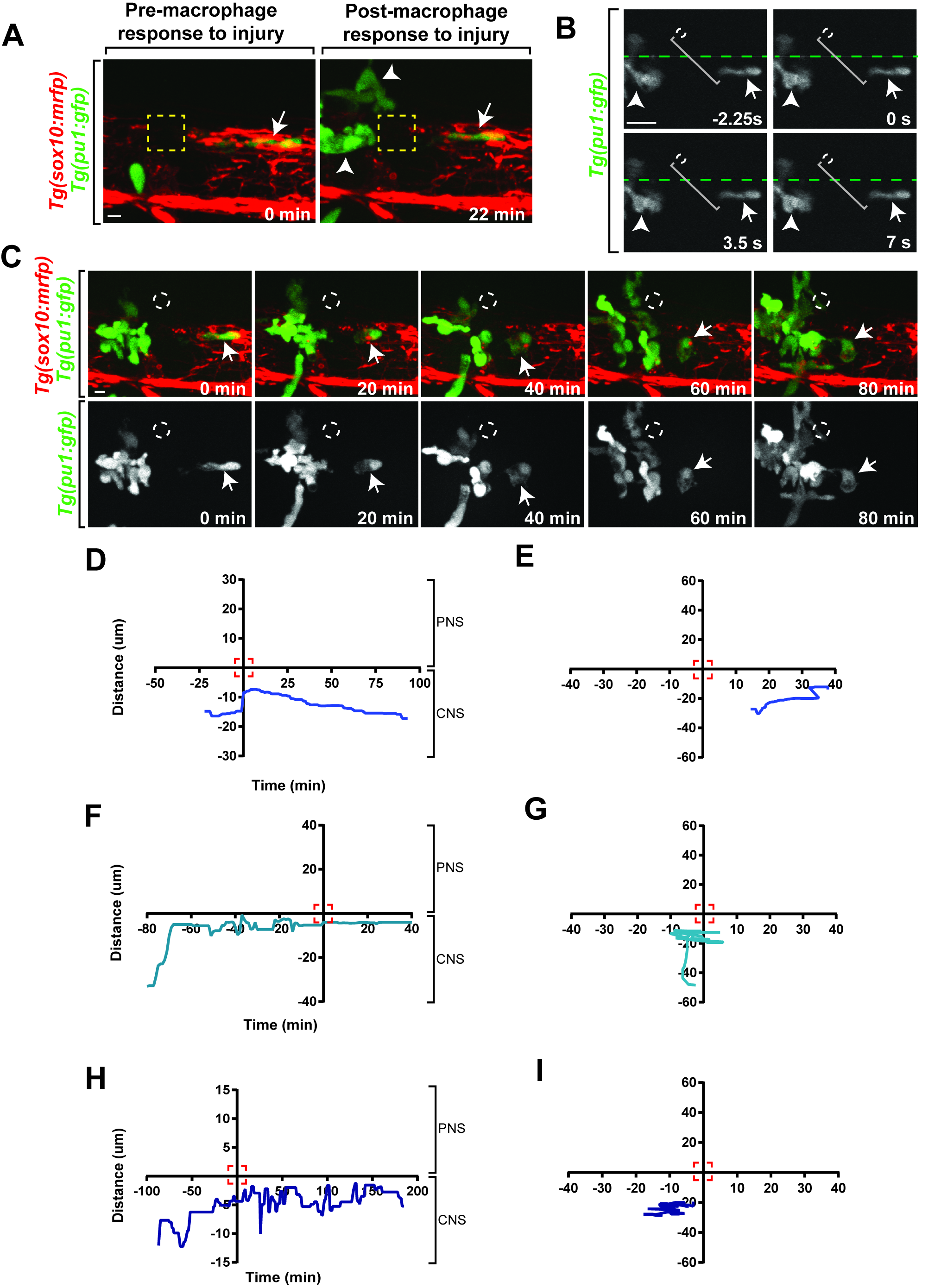

Supplement: S19 Fig — (A) Images from a 30-minute time-lapse movie at 4 dpf in Tg(pu1:gfp);Tg(sox10:mrfp) zebrafish showing the response of macrophages and microglia to the injury site. Arrows indicate microglia. Arrowheads indicate macrophages. Yellow box indicates injury site. (B) Images from a time-lapse ablation window in Tg(pu1:gfp) zebrafish at 4 dpf showing ablation control and no immediate microglial response. (C) Images from a 2-hour time-lapse movie at 4 dpf in Tg(pu1:gfp);Tg(sox10:mrfp) zebrafish showing no microglial response to the site of control ablation. Arrow indicates microglia. White circle indicates site of macrophage ablation. (D, F, H) Migration plots of microglia not responding to site of control ablations. Red box indicated control ablation site. (E, G, I) Quantification of distance microglia traveled pre- and post-control ablation over time. Red box indicates control ablation site. y-axis > 0 indicates cell’s presence in PNS. y-axis < 0 indicates cell’s presence in CNS. x-axis > 0 indicates after ablation. x-axis < 0 indicates before ablation. Scale bar equals 10 μm (A-C). See S23 Data for raw data. CNS, central nervous system; dpf, days post fertilization; PNS, peripheral nervous system. (TIF) [file pbio.3000159.s021.tif]

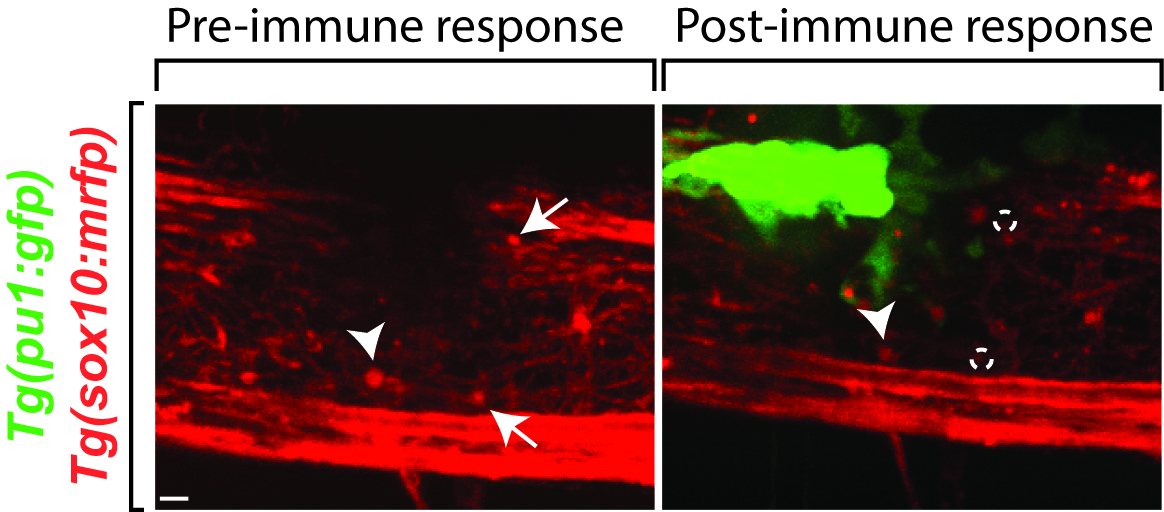

Supplement: S20 Fig — Images from a 24-hour time-lapse movie starting at 4 dpf in Tg(pu1:gfp);Tg(sox10:mrfp) zebrafish post-injury showing macrophage response to injury in animals without microglia. Stills display how debris puncta were determined. Arrows indicate debris that is cleared. Arrowheads indicate debris that is not cleared. Dashed circles represent cleared debris. Scale bar equals 10 μm. dpf, days post fertilization. (TIF) [file pbio.3000159.s022.tif]

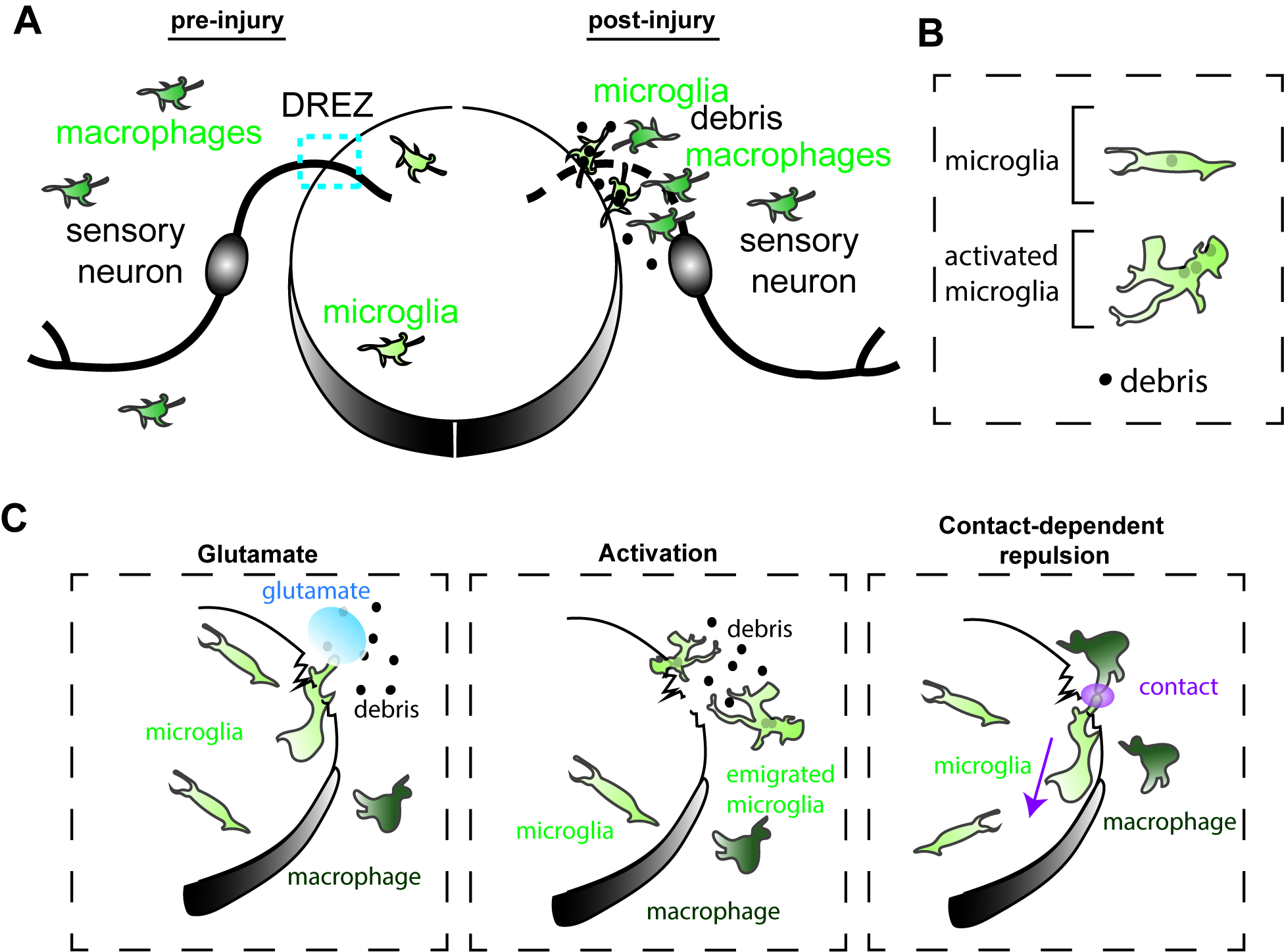

Supplement: S21 Fig — (A) Model describing the observed nature of macrophages and microglia pre- and post-OBPI. (B) Model describing the glutamate-dependent emigration of microglia compared to unaltered microglia that remain in the CNS. (C) Model describing the factors necessary for the efficiency of microglia emigration after OBPI. CNS, central nervous system; OBPI, obstetrical brachial plexus injury. (TIF) [file pbio.3000159.s023.tif]
